# A sapropel-derived *Lactiplantibacillus plantarum* with antimicrobial activity improves intestinal architecture and remodels gut microbiota

**DOI:** 10.3389/fmicb.2026.1881881

**Published:** 2026-07-30

**Authors:** Chongkai Zhai, Junhui Niu, Qinghua Qiao, Hewei Zhang, Fuchao Mao, Chengshui Liao, Zhongyu Liu

**Affiliations:** 1 The 989th Hospital of the Joint Logistics Support Force of Chinese People's Liberation Army, Luoyang, China; 2 College of Food and Drugs, Luoyang Polytechnic, Luoyang, China; 3 Animal Diseases and Public Health Engineering Research Center of Henan Province, Luoyang, China; 4 The Geographical Indication Medicines and Life Health Engineering Research Center of Henan Province, Luoyang, China; 5 College of Animal Science and Technology/Laboratory of Functional Microbiology and Animal Health, Henan University of Science and Technology, Luoyang, China

**Keywords:** antimicrobial activity, biosafety, *Lactiplantibacillus plantarum*, probiotic, sapropel

## Abstract

Identifying safe probiotic strains with potent antimicrobial activity is essential for the development of next-generation microbial therapeutics. Here we report a novel sapropel-derived strain, *Lactiplantibacillus plantarum* (*L. plantarum*) JH-N1, exhibiting antibacterial activity together with favorable probiotic traits. *In vitro* safety evaluation showed no mucin degradation, β-haemolytic activity or cytotoxic effects on Caco-2 cells. *L. plantarum* JH-N1 displayed marked tolerance to simulated gastrointestinal stress, surviving exposure to pH 2 and bile salts, and showed strong cell-surface hydrophobicity, auto-aggregation and co-aggregation with multiple pathogens. The strain also adhered efficiently to Caco-2 cells and remained susceptible to most clinically relevant antibiotics. *In vivo*, acute and subacute toxicity studies in mice revealed no adverse effects on body weight, organ indices, tissue histology, hematological parameters or serum biochemical markers. Notably, *L. plantarum* JH-N1 improved intestinal morphology by promoting villus development and increasing crypt depth in multiple gut segments. Moreover, administration of *L. plantarum* JH-N1 reshaped the gut microbiota, increasing beneficial bacterial populations while reducing potentially harmful taxa. Together, these findings identify *L. plantarum* JH-N1 as a safe and functionally robust probiotic candidate with promise for animal husbandry and biomedical applications.

## 1 Introduction

Antibiotics have transformed modern medicine since the introduction of penicillin in the early twentieth century. However, their overuse use has accelerated antimicrobial resistance, increased adverse drug reactions and promoted the emergence of multidrug-resistant “superbugs” (Lobanovska and Pilla, 2017; Keith and Pamer, 2019; Shi et al., 2021). In livestock production, bacterial pathogens such as *Escherichia coli*, *Salmonella enterica* serovar Typhimurium, *Listeria monocytogenes* and *Staphylococcus aureus* continue to cause major economic losses and threaten animal health (Chlebicz and Śliżewska, 2018). Moreover, the extensive use of antibiotics in animal husbandry contributes to the environmental dissemination of antibiotic-resistance genes, posing an increasing risk to global public health (Abate and Birhanu, 2025). These challenges have intensified the search for sustainable alternatives to conventional antibiotics.

Lactic acid bacteria (LAB) have emerged as promising candidates because of their long history of safe use, antimicrobial activity and functional roles in food and feed fermentation (Chen et al., 2025). Increasing evidence indicates that LAB can serve as viable alternatives to antibiotics for controlling animal infections and improving productivity (Reuben et al., 2019). The Food and Agriculture Organization of the United Nations and the World Health Organization have also recognized the health-promoting potential of appropriately selected probiotic strains (Fontana et al., 2013; Jeong et al., 2016). Specific strains of LAB exert a broad spectrum of advantageous biological properties, including anti-allergic activity, antifungal effects, immunomodulation, anti-viral infection and protection against tissue injury (Peng and Hsu, 2005; Rönnqvist et al., 2007; Barone et al., 2016; Amoranto and Balolong, 2020; Moon et al., 2022; Farahmandi et al., 2023). In addition, several studies have suggested anticancer properties of LAB *in vitro* and in animal models (Garbacz, 2022).

The broad functional potential of LAB has accelerated their application in food, agriculture and pharmaceutical sectors. Regulatory authorities, including the European Food Safety Authority and the United States Food and Drug Administration, have recognized multiple LAB species as safe for use in food systems (Arena et al., 2018). Furthermore, current evidence suggests that the responsible incorporation of LAB into agricultural production systems is unlikely to substantially increase the environmental resistome (Rozman et al., 2022). Nevertheless, each newly isolated LAB strain requires rigorous evaluation of probiotic functionality, genomic safety and toxicological risk before practical application (Pradhan et al., 2019).

Therefore, the objective of the present study was to isolate and characterize a novel LAB strain with potent antibacterial activity and favorable safety properties, thereby providing a scientific basis for its future development in animal husbandry and health-related applications.

## 2 Materials and methods

### 2.1 Isolation, screening, and identification of candidate probiotics

#### 2.1.1 Screening and identification of LAB with antibacterial activity against pathogenic bacteria

Five grams of each soil sample were suspended in 95 mL of sterile phosphate-buffered saline (PBS) and incubated under anaerobic conditions at 37 °C for 48 h. The cultures were then serially diluted and plated onto de Man, Rogosa and Sharpe (MRS) agar supplemented with 1% (w/v) bromocresol purple. Plates were incubated anaerobically at 37 °C for 48 h. Colonies surrounded by yellow halos were selected as presumptive LAB isolates. Following primary screening, candidate isolates were cultured in MRS broth under anaerobic conditions at 37 °C for 48 h. Cultures were centrifuged at 7,426 × *g* at 4 °C for 5 min, and the supernatants were passed through a 0.22-μm membrane filter to obtain cell-free supernatants (CFS). To evaluate antibacterial activity, *S. aureus*, *L. monocytogenes*, *E. coli*, and *S. typhimurium* were used as indicator strains (Supplementary Table S1).

#### 2.1.2 Acid neutralization and hydrogen peroxide removal

Acid neutralization experiment was performed to exclude inhibition caused solely by acidity. Briefly, the pH of CFS was adjusted to neutrality (pH 7), and the antibacterial activity was subsequently detected using the Oxford cup diffusion assay. The pH value of the corresponding organic acid control (a mixture of lactic acid and acetic acid) is adjusted to be identical to the pH of CFS. Catalase (10 mg/mL) was prepared in phosphate buffer (pH 7.0) and added to the CFS at a ratio of 1:100 (v/v). The mixture was incubated at 37 °C for 1 h to eliminate the effect of hydrogen peroxide. Untreated CFS served as the control.

#### 2.1.3 Identification of the candidate strain

The candidate isolates were subjected to Gram staining. Morphological characteristics were examined using a light microscope (Soptop, Shanghai, China) and a scanning electron microscope (Servicebio, Wuhan, China). Genomic DNA was extracted using a bacterial DNA extraction kit. PCR amplification was performed using universal primers 27F (5′-AGAGTTTGATCMTGGCTCAG-3′) and 1492R (5′-GGTTACCTTGTTACGACTT-3′) targeting the *16S rRNA* gene. PCR conditions were as follows: 94 °C for 4 min; 34 cycles of 94 °C for 30 s, 55 °C for 30 s, and 72 °C for 1 min; followed by 72 °C for 10 min. PCR products were analyzed by agarose gel electrophoresis and sequenced commercially. Sequence identity was determined using the BLAST algorithm in the National Center for Biotechnology Information (NCBI) database. A phylogenetic tree was constructed using MEGA11 with the neighbor-joining method.

#### 2.1.4 Determination and assembly of the genome sequence

The purified strain was cultured to the logarithmic phase and subsequently submitted to Bioengineering Co., Ltd. (Shanghai, China) for genome analysis (Kandasamy et al., 2024). FastQC v0.11.2 was used to assess the quality of the raw sequencing reads. SPAdes was employed for *de novo* assembly of the second-generation sequencing data, and GapFiller was used to close gaps in the assembled genome. The NCBI Prokaryotic Genome Annotation Pipeline (PGAP) was used to predict coding sequences and other genomic features. MinCED v0.4.2 was used to predict clustered regularly interspaced short palindromic repeat (CRISPR) sequences. Genome mapping was performed using CGView v1.0 software.

#### 2.1.5 Molecular confirmation of *L. plantarum*

The assembled genome sequence was compared against the NCBI Nucleotide Sequence and Non-Redundant Protein Sequence databases using BLAST to evaluate sequence similarity and infer putative functional attributes. In addition, the *16S rRNA* gene sequence predicted from the genome assembly was aligned against the NCBI database using BLAST. The top 30 homologous 16S rRNA sequences were retrieved and aligned using MAFFT. Phylogenetic trees were subsequently constructed using FastTree, IQ-TREE, and RAxML. This analysis was used to confirm the taxonomic identity and phylogenetic position of the target strain.

#### 2.1.6 Mining of antimicrobial-related genes of *L. plantarum*

Secondary metabolite biosynthetic gene clusters were identified using antiSMASH v5.0. Based on the whole-genome sequencing results, antimicrobial-related genes were further mined using antiSMASH v5.0 and BAGEL4. Transmembrane helices were predicted using TMHMM^1^, and signal peptides were predicted using SignalP^2^. Protein tertiary structures were modeled using SWISS-MODEL^3^.

### 2.2 *In vitro* probiotic properties assessment

#### 2.2.1 Hemolytic activity

Hemolytic activity was determined as previously described (Mulaw et al., 2019). Bacterial cultures were streaked in triplicate on Columbia blood agar medium (BKMAM Biotechnology Co., Ltd., Hunan, China). *S. aureus* served as a positive control and was incubated at 37 °C for 48 h. Subsequently, the plates were examined for the presence of β-haemolysis (clear zones surrounding colonies), α-haemolysis (greenish zones surrounding colonies), or γ-haemolysis (absence of haemolytic zones surrounding colonies).

#### 2.2.2 Mucin degradation

Mucin degradation was assessed as previously described (Fernández et al., 2005). The fundamental composition of the medium consisted of tryptone (Aoboxing Bio-tech Co., Ltd., Beijing, China) 15.0 g, yeast extract (Thermo Fisher Scientific, Shanghai, China) 5.0 g, Tween 80 (Bailun Biotechnology Co., Ltd., Tianjin, China) 1 mL, KH_2_PO_4_ (Chengdu Kelong Chemical Reagent Factory, Sichuan, China) 2.0 g, CH_3_COONa (Chengdu Kelong Chemical Reagent Factory, Sichuan, China) 5.0 g, C_6_H_5_Na_3_O_7_ (Xilong Chemical Co., Ltd., Guangdong, China) 2.0 g, MgSO_4_ (Fengchuan Chemical Reagent Technology Co., Ltd., Tianjin, China) 0.2 g, and MnSO_4_ (Fengchuan Chemical Reagent Technology Co., Ltd., Tianjin, China) 0.05 g. Porcine gastric mucin (Shanghai Genye Biotechnology Co., Ltd., Shanghai, China) and a minimal anaerobic culture medium were utilized. The activated monocultures of probiotic strains were inoculated (10 μL) onto the basic culture medium, both with and without 1% (w/v) glucose, and containing 0.3% (w/v) mucin derived from the porcine stomach. These cultures were then subjected to anaerobic incubation at 37 °C for 48 h. After incubation, the cultures were stained with 0.1% amido black in 3.5 mol/L acetic acid for 30 min. Subsequently, amido black was washed with 1.2 mol/L acetic acid until a mucin lysis zone (a discolored halo) became visible around the colony.

#### 2.2.3 Cytotoxic activity of candidate strain and CFS toward Caco-2 cells

The cytotoxicity was assessed in accordance with the guidelines provided by the Cell Counting Kit 8 (CCK-8, Beyotime Biotechnology, Shanghai, China). Caco-2 cells were cultured in a complete medium and seeded at a density of 5 × 10^5^ cells per well in a 96-well plate. The cells were then incubated at 37 °C in 5% CO_2_ for 24 h to allow for attachment. *L. plantarum* was cultivated for 12 h, and plate counting was performed. The strain was added to Caco-2 cells at multiplicities of infection (MOI) of 10, 20, 50, and 100. CFS was prepared at concentrations of 5, 10, 20, and 50% (v/v) and added to Caco-2 cells, which were incubated at 37 °C and 5% CO_2_ for 24 h. Subsequently, 100 μL of Dulbecco’s Modified Eagle’s Medium (DMEM) and 10 μL of CCK-8 were added to each well, and the mixture was incubated at 37 °C for 2 h. The absorbance at OD450 nm was measured using a Spectrophotometer (Dequan Xingye Trading Co., Ltd., Henan, China). Cell viability (%) was calculated using the formula provided below:

$$\text{Cell viability}\;(\%)=[(\mathrm{Aa}-\mathrm{Ab})/(\mathrm{Ac}-A_{b})]\times 100\%$$

The experimental group is represented as *A*_a_, which refers to the cell viability of candidate strains or CFS added to Caco-2 cells. The control group is represented as *A*_c_, consisting of Caco-2 cells at 100% viability. The blank group, denoted as *A*_b_, received only DMEM.

#### 2.2.4 Tolerance of acid and bile salts

The acid and bile salt tolerance of LAB was assessed using the previously described method (Śliżewska et al., 2021). The pH of the MRS broth was adjusted to 2, 3, and 4. LAB was resuspended in the acidic solutions described above at a concentration of 10^8^ CFU/mL, and the untreated MRS suspension served as a control. Aliquots were taken at intervals of 0, 1, 2, 3, and 4 h, serially diluted, and plated onto MRS agar. The plates were incubated anaerobically at 37 °C for 48 h, and viable counts were obtained. MRS broth supplemented with 0.3, 0.5, and 1% bile salt (Sigma Aldrich, Missouri, United States) was prepared, with a preparation without bile salt serving as the control. The LAB was resuspended in the solution mentioned above at a concentration of 10^8^ CFU/mL. Aliquots were taken at 0, 1, 2, 3, and 4 h, serially diluted, and plated onto MRS agar. The plate count method was employed to determine the number of LAB colonies on MRS agar. The survival rate was calculated using the following equation:

Survival rate(%)=[Ax/A0]×100%

The number of bacteria after treatment with acid or bile salts is expressed in CFU/mL and denoted as *A*_x_, while the number of untreated bacteria, also expressed in CFU/mL, is denoted as *A*_0_.

#### 2.2.5 Cell surface hydrophobicity

The cell surface hydrophobicity was determined as previously described (Niederle et al., 2019), with some improvements. The candidate strains were centrifuged at 7426 *g* for 5 min. Subsequently, the suspension of LAB cells was collected and washed twice with PBS to achieve a concentration of 10^8^ CFU/mL. A mixture comprising 1 mL of xylene and 3 mL of the cell suspension was prepared, vigorously mixed, and vortexed for 2 min. The resulting mixture was then incubated at room temperature for 2 h, forming a two-phase system with the organic phase separating from the aqueous phase. The organic phase was removed. The absorbance of the aqueous phase was measured at a wavelength of 600 nm, with PBS serving as the control. The degree of bacterial cell surface hydrophobicity was categorized as low (0 ~ 29%), medium (30 ~ 59%), or high (60 ~ 100%).

The cell surface hydrophobicity (%) was calculated using the formula:

Cell surface hydrophobicity(%)=[(A0−A1)/A0]×100%

Here, A_0_ is the initial OD600 of LAB and A_1_ is OD600 of the water phase after the incubation.

#### 2.2.6 Aggregation and co-aggregation assay with pathogens

The aggregation assay was conducted following a previously described methodology (Malfa et al., 2023). The suspension of LAB was obtained by centrifugation at 7,246 *g* for 5 min, followed by washing and re-suspension in PBS to achieve a concentration of 10^8^ CFU/mL. The LAB cell suspension was subjected to vortex shaking for 20 s and then incubated at 37 °C. Subsequently, the absorbance of the upper suspension (100 μL) was measured at 600 nm using a spectrophotometer at 0, 2, 4, 6, 10, and 24 h. The rate of auto-aggregation was determined using the following calculation:

Auto−aggregation(%)=[(A0−At)/A0]×100%

Here, A_0_ is the absorbance at time 0 h, while A_t_ is the absorbance at 2, 4, 6, 10, or 24 h.

For the co-aggregation assay based on a previously described method (Śliżewska et al., 2021), the cell suspensions were prepared the same way as for the auto-aggregation test. Equal volumes of monocultures of LAB and pathogenic bacterial suspensions (*S. aureus*, *L. monocytogenes*, *E. coli*, and *S. typhimurium*) were mixed by vortexing for 20 s. After 24 h of incubation at ambient temperature, the absorbance of the upper suspension was measured at 600 nm. The percentage of co-aggregation was calculated according to a previously established method (Malfa et al., 2023).

Co−aggregation(%)=[1−2×−Amix/(ALAB+APath)]×100%

Here, A_LAB_ and A_Path_ represent the initial OD600 of LAB and pathogenic bacteria, respectively, while A_mix_ denotes the OD600 of the mixture after incubation.

#### 2.2.7 Adhesion assay to Caco-2 cells

The Caco-2 cells were cultured (5 × 10^5^ cells/mL) in 12-well microplates (Biofil, Guangzhou, China) in 5% CO_2_ at 37 °C. The candidate strain was harvested during the mid-logarithmic phase and added to the cells at an MOI of 100 at 37 °C for 2 h. Each experiment was performed in triplicate. After incubation, the unbound microorganisms were aspirated, and the plate was washed twice with sterile PBS. Then, cells containing adherent bacteria were lysed with 1 mL of 0.1% (w/v) Triton-100 (Solarbio, Beijing, China). After 15 min of incubation, the solution containing the released bacteria was collected, serially diluted, and plated on MRS agar. The adhesion rate was calculated according to the formula:

Adhesion ability(%)=[Ax/At]×100%

Here, A_x_ is the initial bacteria count, and A_t_ is the bacterial adhesion count.

After removing non-adherent microorganisms, the wells were rinsed with PBS, and the Caco-2 cells were fixed with 4% (v/v) paraformaldehyde for 15 min. After air-drying, the cells were stained using Gram staining. The adhesion was observed by microscopy. This procedure was adapted from a previously described methodology (Wang et al., 2022).

#### 2.2.8 Susceptibility test

The antibiotic sensitivity of LAB was assessed using the disk diffusion method as recommended by the National Committee for Clinical Laboratory Standards. Commonly used veterinary antibiotics were employed to determine antibiotic susceptibility. LAB in the logarithmic phase, with approximately 10^8^ CFU/mL, was utilized for the experiment. The LAB suspensions were seeded onto MRS agar plates using a glass rod. Antibiotic-impregnated disks were placed on the seeded plates and incubated at 37 °C for 24 h. The assay was conducted in triplicate, and the size of the inhibition zone was measured to determine the antibiotic susceptibility.

### 2.3 *In vivo* probiotic properties assessment

#### 2.3.1 Acute toxicity test

To evaluate the acute toxicity of *L. plantarum*, mice were acclimated and divided into a control group and experimental groups, with 10 mice in each group. The control group was administered oral saline, while the three experimental groups were gavaged with *L. plantarum* at doses of 1 × 10^9^, 1 × 10^10^, and 1 × 10^11^ CFU, respectively. All animal procedures were reviewed and approved by the Laboratory Animal Welfare and Ethical Committee of the Henan University of Science and Technology (approval no. HAUST-026-M0429080). Experimental protocols were performed in compliance with the institutional guidelines for the care and use of laboratory animals established by the Henan University of Science and Technology. Daily observations were carried out for 7 days to monitor clinical toxicity symptoms and mortality. Blood samples were collected after 7 days and cultured anaerobically in 1% MRS medium. If bacterial growth occurred, colonies were identified by PCR. The acute oral toxicity test experiment was performed in accordance with the established protocol (Deng et al., 2023).

#### 2.3.2 Subchronic toxicity test

After a period of acclimation, the control group received equal amounts of saline via gavage. The experimental group received *L. plantarum* via gavage at doses of 1 × 10^7^, 1 × 10^9^, and 1 × 10^11^ CFU once daily. Throughout the 28-day gavage period, the mice were observed daily for clinical signs. Additionally, their weights were recorded weekly (Samtiya et al., 2020). On day 28, the mice were weighed, and blood samples were collected to assess routine blood and biochemical indexes. Subsequently, the mice were euthanized, and an anatomical examination was performed to determine the organ indices of the heart, liver, spleen, lungs, and kidneys. Simultaneously, a pathological histological examination was conducted on the heart, liver, spleen, lungs, kidneys, and intestines. Homogenates of aseptically collected organ tissues were cultured anaerobically in 1% MRS medium. Bacterial growth, if any, was identified by PCR.

#### 2.3.3 Intestinal flora

Based on the study of Wiredu Ocansey et al. (2023) on the intestinal flora of mice, fecal samples were collected after the 28-day subchronic toxicity test and immediately transported on dry ice to Oebiotch (Shanghai, China). High-throughput sequencing was carried out using the Illumina Hiseq 2500 (PE250) system. The original image data files were transformed into original paired-end sequences through base calling analysis. Quality control on the original sequences was performed using Utilizing Trimmatic (v 0.35), FLASH (v 1.2.11), Split_ Libraries (v 1.8.0), and UCHIME (v 2.4.2) to obtain high-quality sequences. Using DADA2 from QIIME2, the sequences were denoised, chimeras were removed, and then deduplicated. Each deduplicated sequence was referred to as an Amplicon Sequence Variant (ASV). Diversity analyses, including the Chao1 index, Coverage index, Simpson index, and Shannon index, as well as microbial composition analysis, were performed. Additionally, the different species of gut microbiota in four groups of mice were compared.

### 2.4 Statistical analysis

The experimental data were presented as the mean ± standard deviation. The comparison of the mean values was performed by one-way analysis of variance (ANOVA) and Tukey’s test using the IBM SPSS Statistics 25 software (SPSS Inc., Chicago, United States). *p* < 0.05 was considered statistically significant. The data were further processed, and figures were generated using GraphPad Prism 8.0.1 (GraphPad Inc., San Diego, California, United States) and Biorender software (Biorender Inc., Toronto, Canada).

## 3 Results

### 3.1 Isolation, screening, and identification of LAB with antibacterial activity

#### 3.1.1 Isolation of antibacterial LAB

A total of 135 LAB isolates obtained from 179 soil samples were screened (Supplementary Figure S1). Among these isolates, 40 strains exhibited antibacterial activity against pathogenic bacteria, whereas 15 strains displayed broad-spectrum antibacterial activity (Supplementary Table S2). Among them, strain JH-N1 showed particularly strong inhibitory activity and was therefore selected for further study.

#### 3.1.2 Acid neutralization and hydrogen peroxide removal

**Figure 1:**
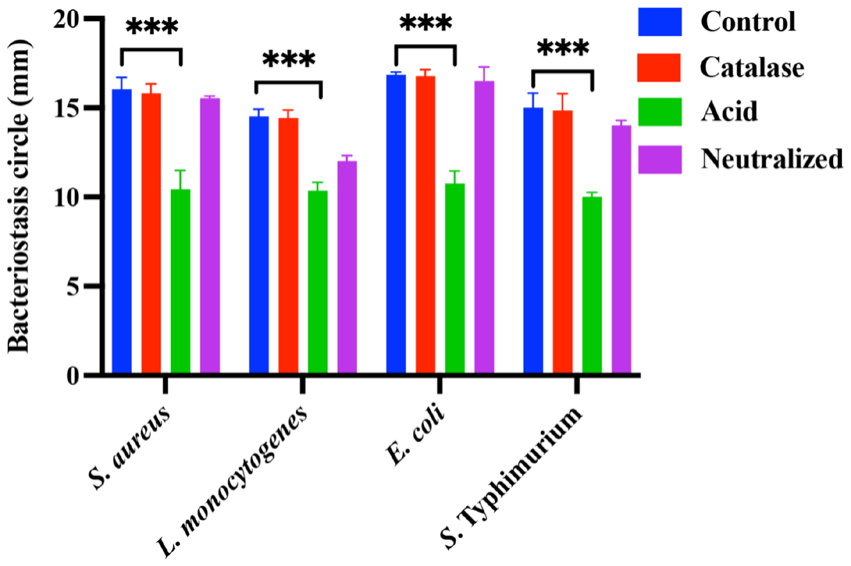
Effects of acid neutralization and hydrogen peroxide removal on the antibacterial activity of the LAB strain with strong inhibitory potential. Control, untreated CFS of JH-N1; neutralized: acid-neutralized CFS of JH-N1; Catalase, CFS of JH-N1 treated with catalase; Acid, organic acid solution containing lactic acid and acetic acid. ***p < 0.001.

The CFS of strain JH-N1 showed significantly greater inhibitory activity against pathogenic bacteria than the corresponding organic acid control. A comparable antibacterial efficacy was observed between acid-neutralized CFS and untreated CFS. After treatment of the CFS with catalase for 1 h, no significant reduction in antibacterial activity was observed compared with the untreated CFS (Figure 1). These results indicate that extracellular metabolites other than organic acids or hydrogen peroxide contributed to the antibacterial activity of strain JH-N1.

#### 3.1.3 Identification of antibacterial strain

**Figure 2:**
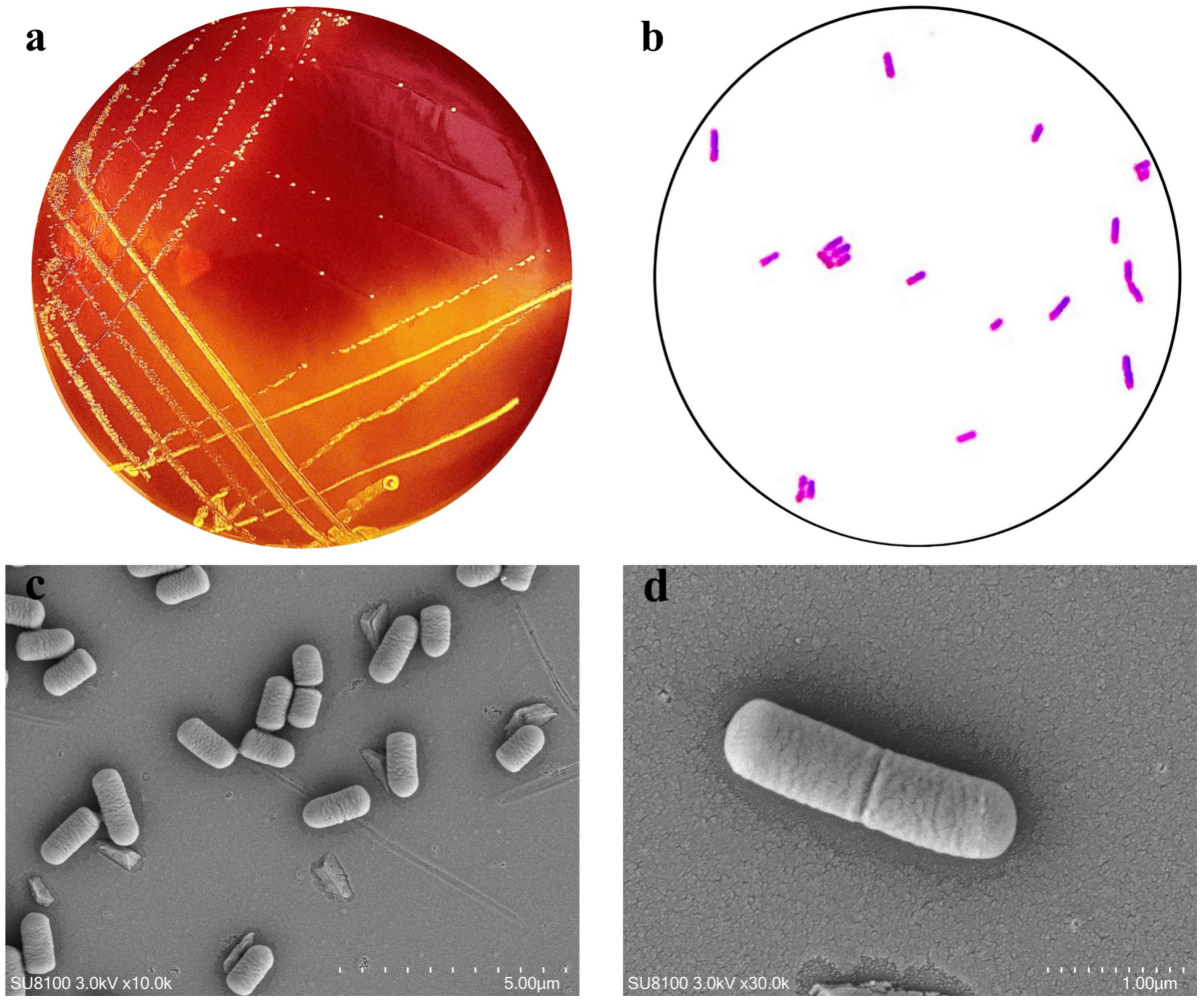
Morphological identification of *L. plantarum* JH-N1. **(a)** Colony morphology of strain JH-N1 cultured on MRS agar supplemented with 1% bromocresol purple. **(b)** Gram-stained micrograph of *L. plantarum* JH-N1 (1,000× magnification). **(c,d)** Scanning electron micrographs of *L. plantarum* JH-N1.

The JH-N1 strain was cultured on MRS agar supplemented with 1% bromocresol purple. Single colonies surrounded by yellow halos, indicating acid production, were selected. These colonies were of uniform size and displayed similar morphology (Figure 2a). Gram staining was performed on strain JH-N1, and the samples were examined under a microscope at 1,000× magnification. The observed rod-shaped cells exhibited a blue-purple color, confirming that the isolate was Gram-positive (Figure 2b). Scanning electron microscopy was used to further examine cellular morphology, and the results were consistent with those obtained by Gram staining (Figures 2c,d). Genomic DNA from strain JH-N1 was extracted and subjected to PCR amplification, yielding a specific amplicon of approximately 1,479 bp (Supplementary Figure S2). Furthermore, analysis of the 16S rRNA gene sequence revealed 99.93% identity to *L. plantarum* WSJ-11 (Supplementary Figure S3). Based on these findings, strain JH-N1 was identified as *L. plantarum*.

#### 3.1.4 Genomic information of *L. plantarum* JH-N1

**Figure 3:**
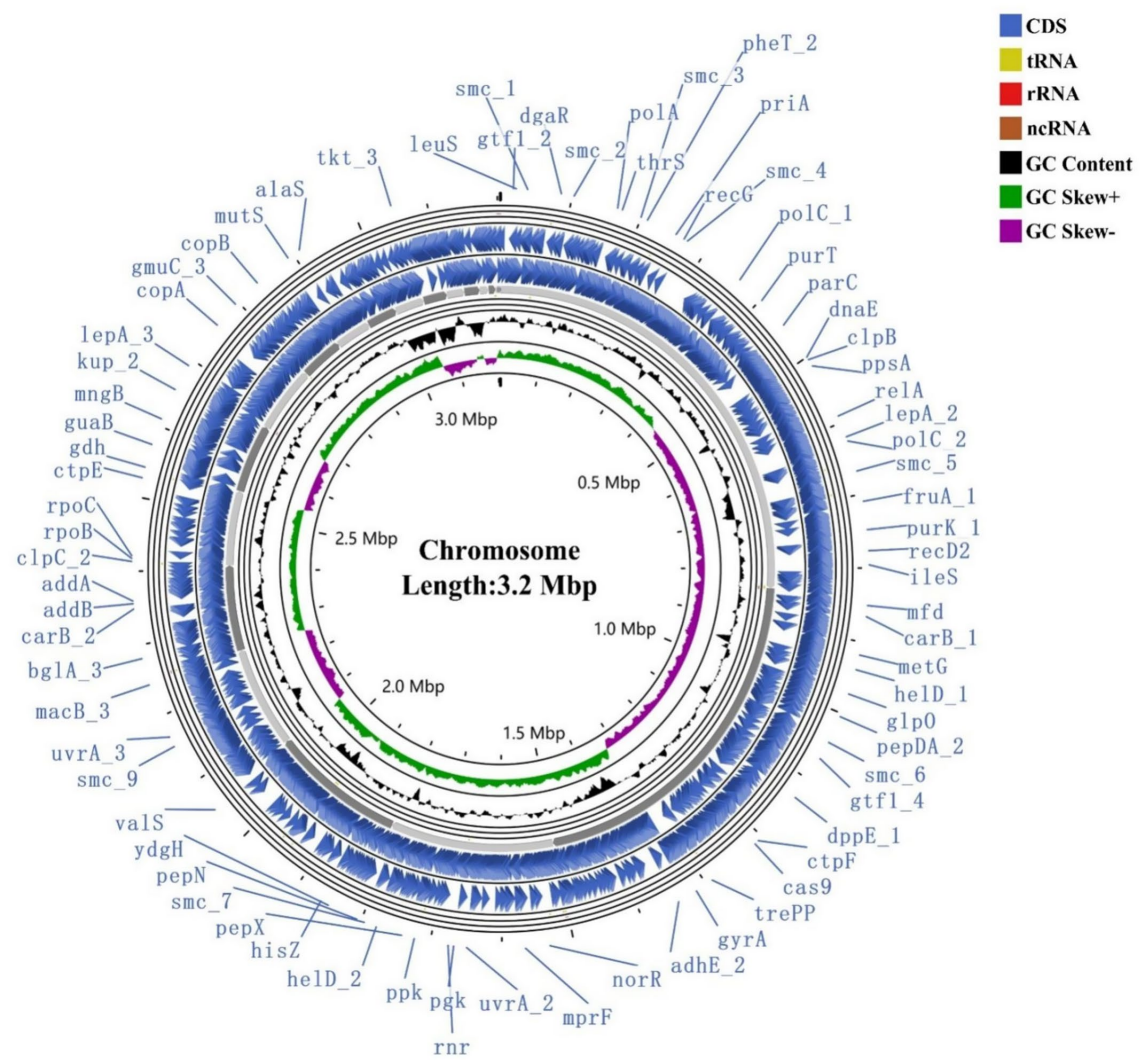
Complete genome map of *L. plantarum* JH-N1. Circular chromosome features predicted from Illumina sequencing data are visualized using CGView.

The genome of *L. plantarum* JH-N1 was subjected to Illumina sequencing, followed by quality assessment and data filtering. High-quality second-generation sequencing reads were assembled using SPAdes. The whole genome sequence of *L. plantarum* JH-N1 has been deposited in the NCBI GenBank database under the accession number SUB16153499. Genome survey analysis was performed using sequencing-based K-mer analysis of low-depth short-insert libraries, enabling rapid estimation of the genomic characteristics of the strain. CGView was used to generate the circular genome map (Figure 3). The results showed that the chromosome size of *L. plantarum* JH-N1 was 3,204,278 bp, with a GC content of 44.5%. The predicted numbers of CRISPR loci, genomic islands, and prophages were 3, 0, and 0, respectively. In addition, the genome contained 7 rRNA genes, 64 tRNA genes, and 1 ncRNA gene (Table 1).

**Table 1:** Genomic information of *L. plantarum* JH-N1.

Indicator	Number or content
Chromosome (bp)	3,204,278
G + C content of chromosome (%)	44.5%
Coding gene numbers	2,998
Total length of coding genes (bp)	2,696,097
Total rRNA numbers	7
Total length of rRNA (bp)	4,774
Total tRNA numbers	64
Total length of tRNA (bp)	4,982
Total ncRNA numbers	1
Total length of ncRNA (bp)	369
Total feature numbers	3,070
Total length of feature	2,706,222
CRISPRs	3
Pseudogene numbers	0
Genomic islands	0
Plasmid	0

#### 3.1.5 Mining of bacteriocins from *L. plantarum* JH-N1

**Figure 4:**
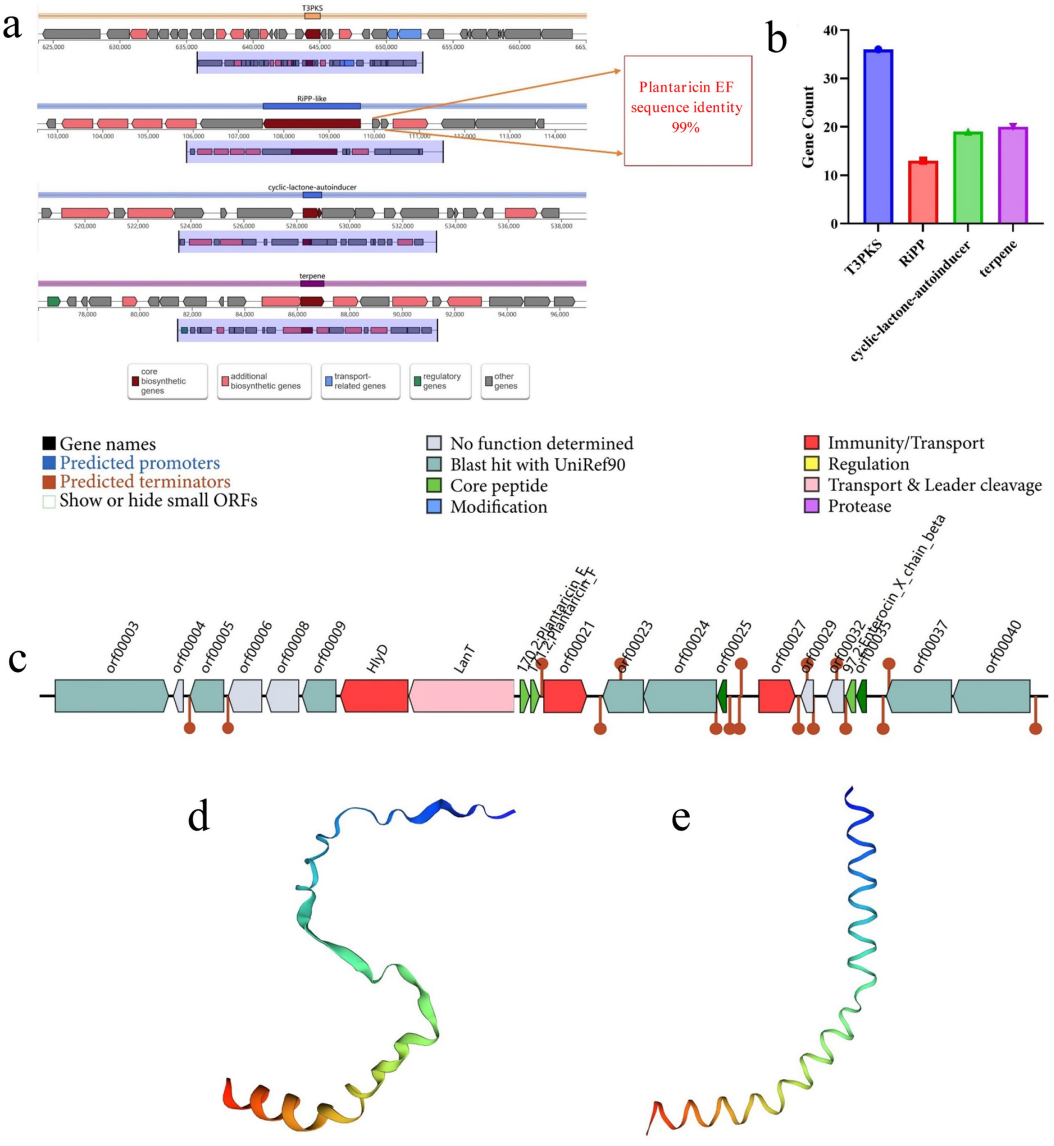
Mining of bacteriocins from *L. plantarum* JH-N1. **(a)** Prediction of secondary metabolite biosynthetic gene clusters in *L. plantarum* JH-N1 using antiSMASH. **(b)** Number and distribution of predicted secondary metabolite-related genes. **(c)** Major bacteriocin biosynthetic gene clusters identified using the BAGEL4 database. **(d,e)** Predicted tertiary structures of the putative bacteriocins.

The secondary metabolite biosynthetic potential of *L. plantarum* JH-N1 was analyzed using antiSMASH, and the results are shown in Figures 4a,b. Region 2 was identified as a ribosomally synthesized and post-translationally modified peptide (RiPP) gene cluster, which may encode peptides with antibacterial activity. Further analysis of the Region 2 cluster identified two putative bacteriocin genes, designated ctg00002_00941 and ctg00002_00942 (Table 2). The predicted core peptides both belonged to class IIb bacteriocins and shared 99% sequence identity with plantaricin EF. In addition, bacteriocin-associated gene clusters were analyzed using BAGEL4, which mines and visualizes RiPPs and bacteriocin biosynthetic loci in prokaryotic genomes. As shown in Figure 4c, two putative bacteriocins were also predicted, consistent with the antiSMASH results. The predicted tertiary structures of the candidate bacteriocins from *L. plantarum* JH-N1 were subsequently modeled and are shown in Figures 4d,e.

**Table 2:** *Lactiplantibacillus plantarum* JH-N1 bacteriocins prediction.

Gene ID	Start	End	Sequence	Relative molecular mass	Theoretical PI
ctg00002_00941	109,969	110,139	MLQFEKLQYSRLPQKKLAKISGGFNRGGYNFGKSVRHVVDAIGSVAGIRGILKSIR	6191.30	11.11
ctg00002_00942	110,164	110,322	MKKFLVLRDRELNAISGGVFHAYSARGVRNNYKSAVGPADWVISAVRGFIHG	5732.61	10.52

#### 3.1.6 Mining of virulence and tolerance genes of *L. plantarum* JH-N1

The genome of *L. plantarum* JH-N1 was screened against VFDB to assess its potential pathogenicity. Only one putative virulence-associated gene, *clpP*, was identified (Supplementary Table S3). However, *clpP* is widely distributed among Gram-positive bacteria and is primarily involved in protein quality control, stress adaptation, and cellular homeostasis rather than direct virulence expression. No experimentally validated virulence determinants typically associated with pathogenic bacteria were detected, suggesting a favorable genomic safety profile for *L. plantarum* JH-N1. To further evaluate antimicrobial resistance potential, the genome was analyzed using CARD. Four putative resistance-associated genes were predicted, all mainly related to multidrug efflux systems (Supplementary Table S4). Such genes are commonly found in non-pathogenic lactic acid bacteria and may contribute to intrinsic resistance or environmental adaptation, although their functional significance in *L. plantarum* JH-N1 requires further verification. Genes associated with environmental tolerance and probiotic fitness were also identified. These included 10 universal stress protein genes, six heat tolerance-related genes, three cold tolerance-related genes, one bile salt tolerance-related gene, 13 pH resistance-related genes, and 16 antioxidant-related genes (Supplementary Table S5). The presence of these functional genes suggests that *L. plantarum* JH-N1 possesses strong adaptive capacity under gastrointestinal and environmental stresses. Overall, the absence of confirmed virulence determinants together with the presence of multiple tolerance-associated genes indicates that *L. plantarum* JH-N1 has a favorable safety profile and promising potential for probiotic application and industrial development.

### 3.2 *In vitro* evaluation of potential probiotic properties

#### 3.2.1 Hemolytic activity

**Figure 5:**
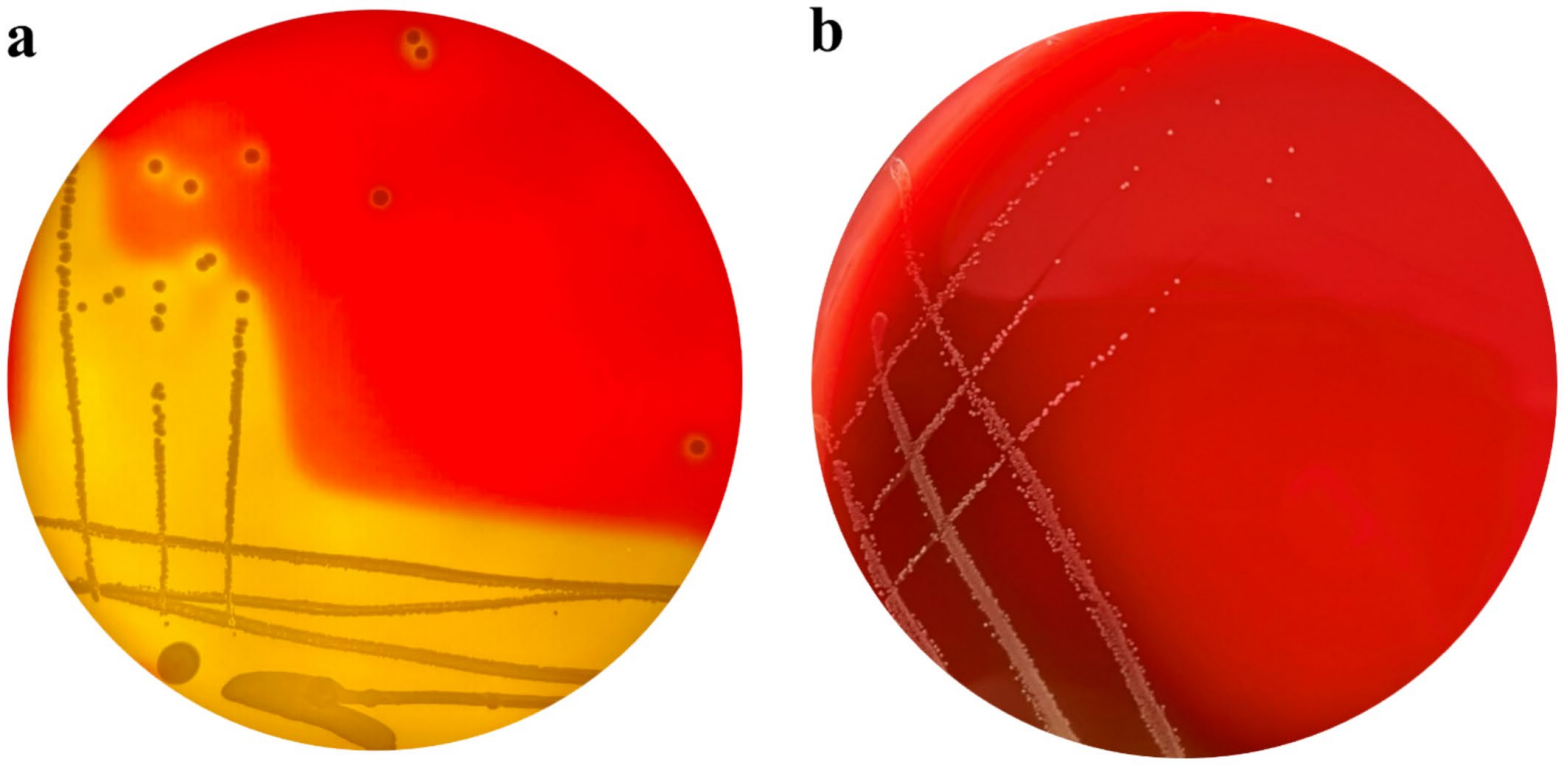
Haemolytic activity of *L. plantarum* JH-N1 on Columbia blood agar plates. **(a)** *S. aureus* (positive control). **(b)** *L. plantarum* JH-N1.

Green halos were observed around colonies of *S. aureus* grown on Columbia blood agar plates, indicating α-haemolytic activity. In contrast, *L. plantarum* JH-N1 exhibited no detectable haemolysis (Figure 5).

#### 3.2.2 Mucin degradation

**Figure 6:**
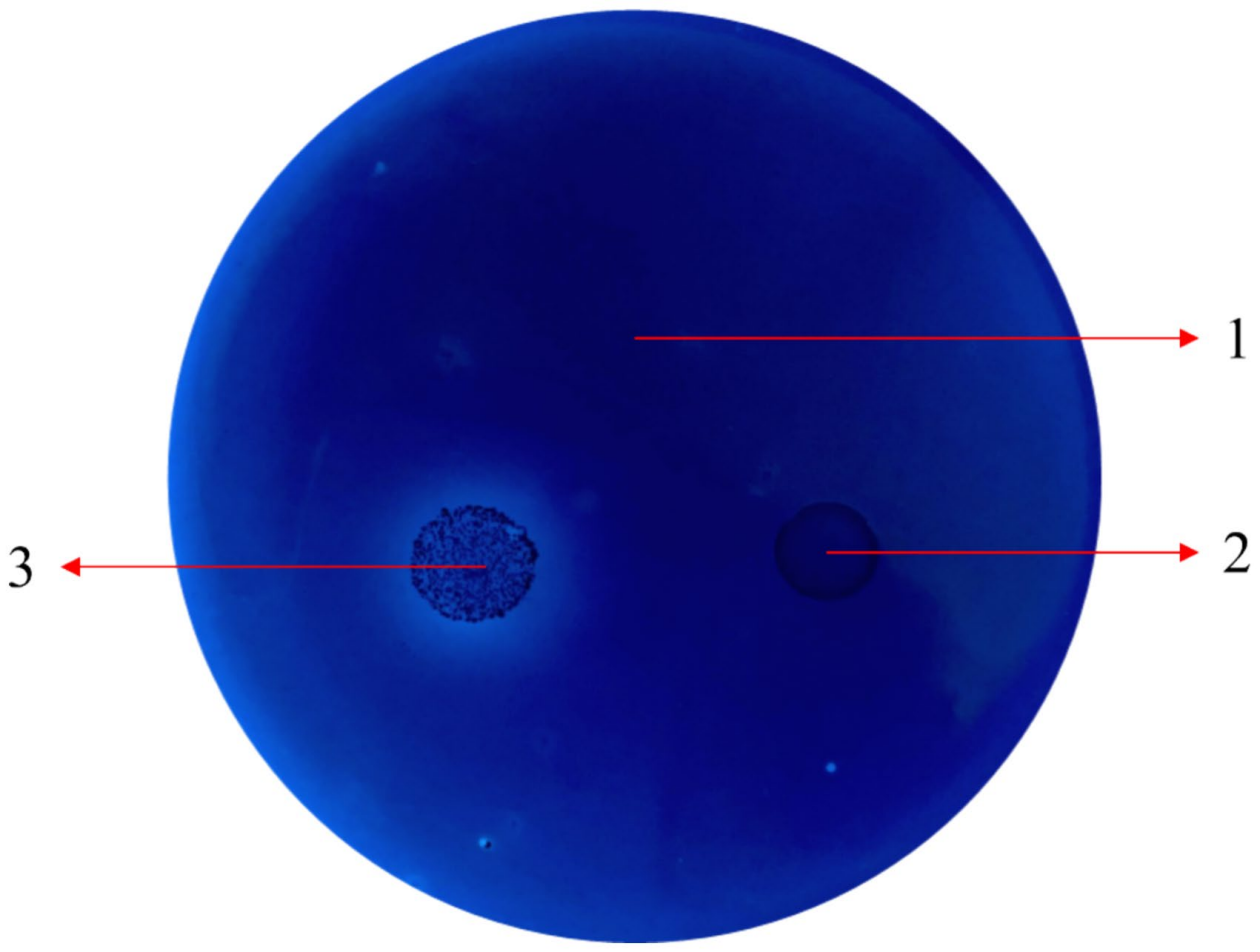
Mucin degradation assay on agarose plates. (1) Culture medium control. (2) *L. plantarum* JH-N1. (3) Fecal microbiota (positive control).

Zones of lysis surrounding colonies were considered indicative of mucolytic activity. A distinct halo of hydrolyzed mucin was observed around the fecal microbiota colonies. In contrast, no discolored halos were detected around *L. plantarum* JH-N1, indicating that this strain lacked detectable mucin-degrading activity (Figure 6).

#### 3.2.3 Cytotoxic activity of candidate strain and CFS toward Caco-2 cells

**Figure 7:**
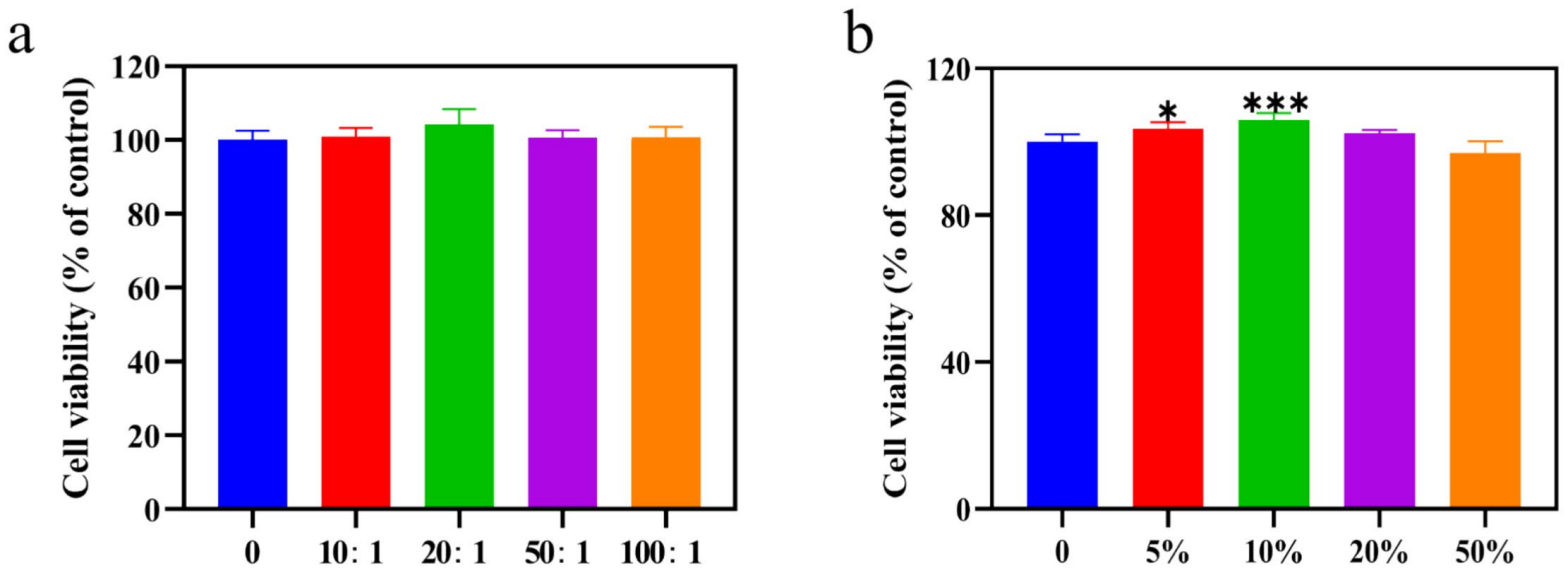
Viability of Caco-2 cells following exposure to *L. plantarum* JH-N1 or its CFS. **(a)** Cell viability after infection with *L. plantarum* JH-N1 at different MOI. **(b)** Cell viability in the presence of different concentrations of CFS. Untreated Caco-2 cells without bacterial/CFS were set as the baseline. *p < 0.05, ***p < 0.001.

Exposure of Caco-2 cells to *L. plantarum* JH-N1 at MOI of 10, 20, 50 and 100 did not significantly affect cell viability (Figure 7a). In addition, the effects of CFS on Caco-2 cells were evaluated at concentrations of 5, 10, 20 and 50% (v/v) for 12 h. Notably, CFS showed no cytotoxicity and significantly increased cell viability at the lower concentrations of 5 and 10% (Figure 7b). These findings further support the safety and probiotic potential of *L. plantarum* JH-N1, providing a basis for subsequent adhesion assays.

#### 3.2.4 Tolerance of acid and bile salts

**Figure 8:**
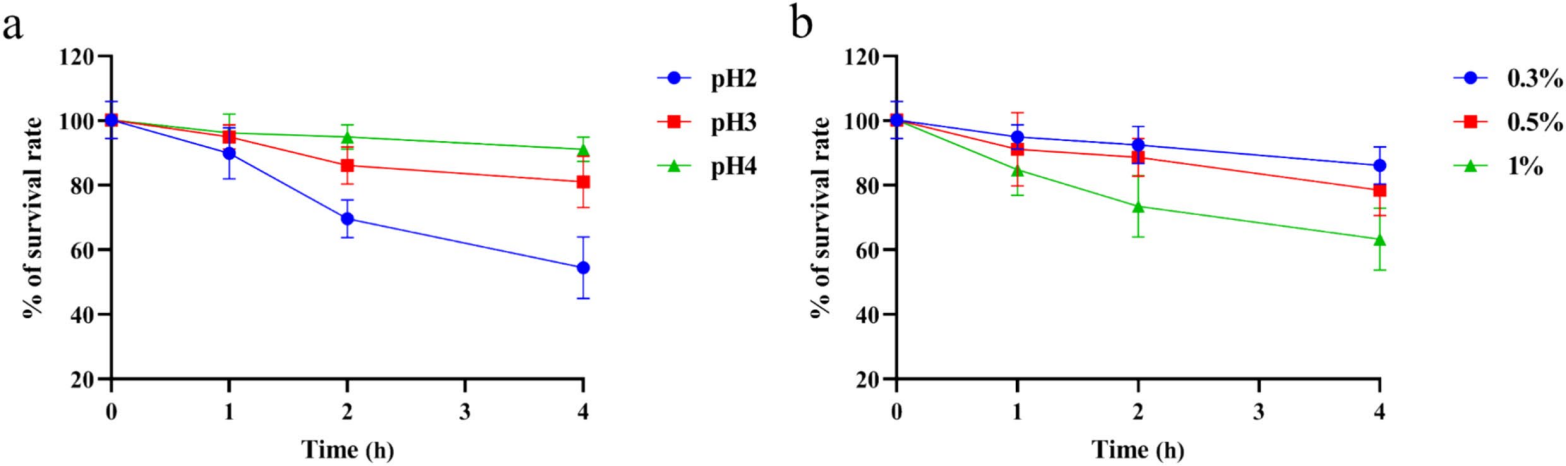
Survival rate of *L. plantarum* JH-N1 after incubation at 37 °C under different acid and bile salt conditions. **(a)** Survival rate at different pH values. **(b)** Survival rate in different bile salt concentrations.

The survival rates of *L. plantarum* JH-N1 after 4 h of incubation at pH 3.0 and pH 4.0 were 81 and 91%, respectively. Notably, the strain retained a survival rate of 54% even at pH 2.0 (Figure 8a). In addition, *L. plantarum* JH-N1 remained viable in the presence of 0.3, 0.5 and 1.0% (w/v) bile salts, with survival rates exceeding 60% after 4 h of incubation (86, 78, and 63%, respectively) (Figure 8b). These results indicate strong tolerance of the strain to simulated gastrointestinal stress conditions.

#### 3.2.5 Cell surface hydrophobicity

**Figure 9:**
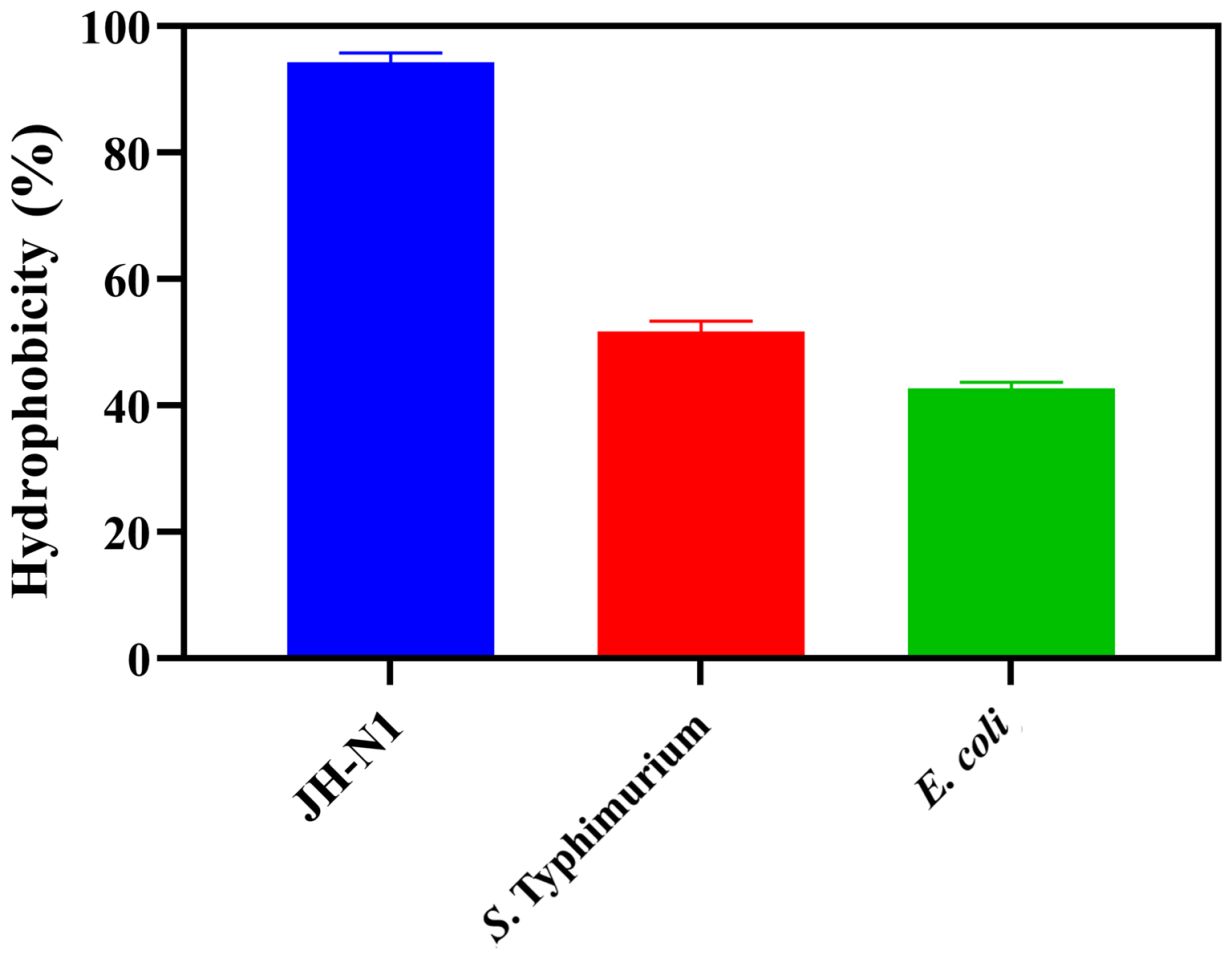
Cell surface hydrophobicity of *L. plantarum* JH-N1. Hydrophobicity was determined by the xylene adhesion assay and compared with indicator strains.

Cell surface hydrophobicity is widely regarded as an important factor associated with bacterial colonization and adhesion within the intestinal environment. *L. plantarum* JH-N1 exhibited a high hydrophobicity value of 94.29%, whereas *S. typhimurium* and *E. coli* showed moderate hydrophobicity values of 51.67 and 42.72%, respectively (Figure 9). These results suggest a strong adhesion potential of *L. plantarum* JH-N1.

#### 3.2.6 Auto-aggregation and co-aggregation with pathogens

**Figure 10:**
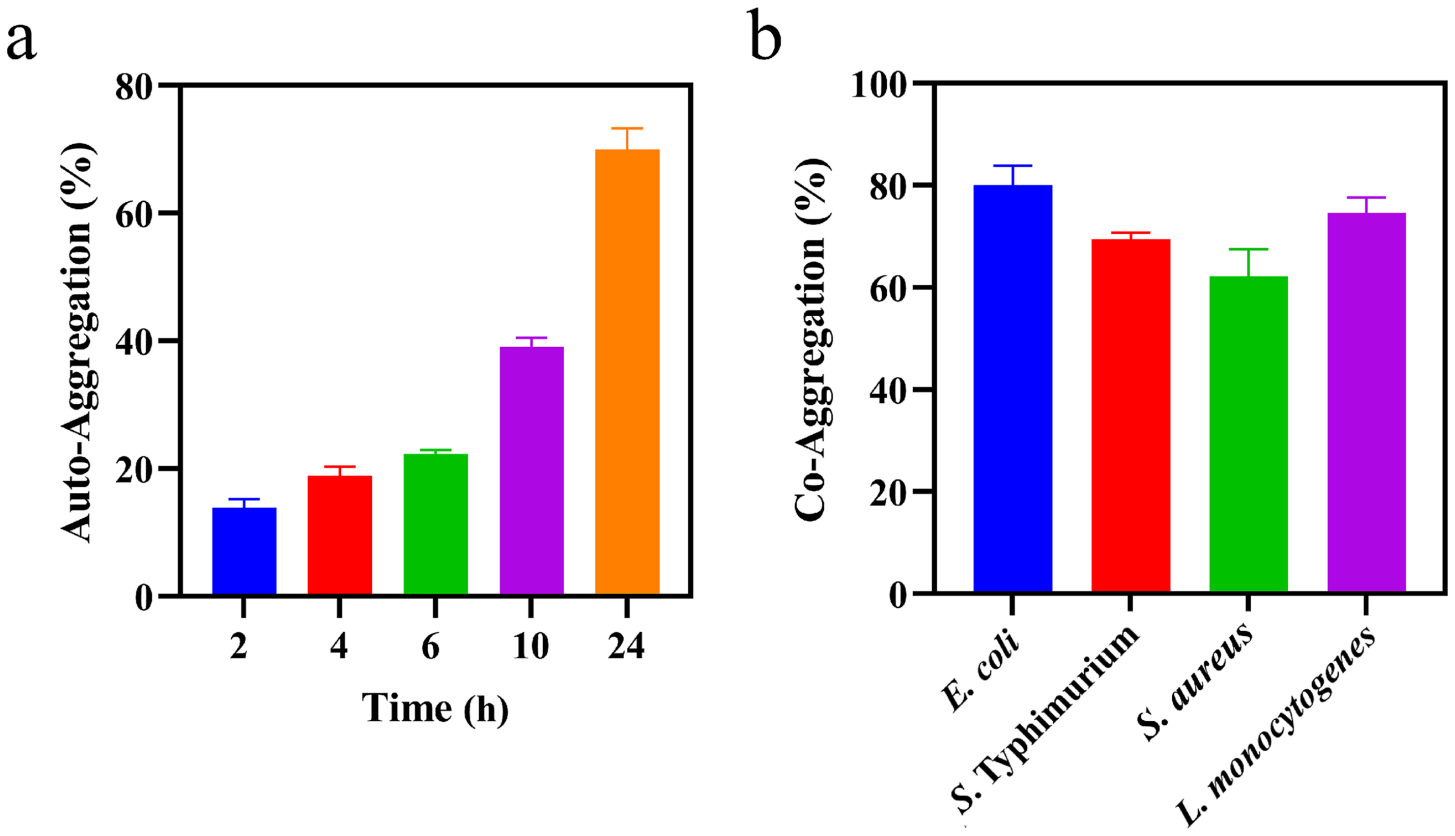
Auto-aggregation and co-aggregation properties of *L. plantarum* JH-N1. **(a)** Auto-aggregation (%) of *L. plantarum* JH-N1 measured at different time points. **(b)** Co-aggregation (%) of *L. plantarum* JH-N1 with pathogenic bacteria measured at different time points.

The auto-aggregation ability of *L. plantarum* JH-N1 was evaluated at 2, 4, 6, 10, and 24 h, as shown in Figure 10a. After 24 h, JH-N1 exhibited an auto-aggregation rate of 69.94%. Co-aggregation assays demonstrated that *L. plantarum* JH-N1 was able to aggregate with all four tested pathogens (Figure 10b). Among them, *E. coli* showed the highest co-aggregation rate (80.07%), whereas *S. aureus* exhibited the lowest value (62.20%). These findings indicate a strong capacity of *L. plantarum* JH-N1 to interact with and potentially inhibit pathogenic bacteria through aggregation-mediated exclusion mechanisms.

#### 3.2.7 Adhesion assay to Caco-2 cells

**Figure 11:**
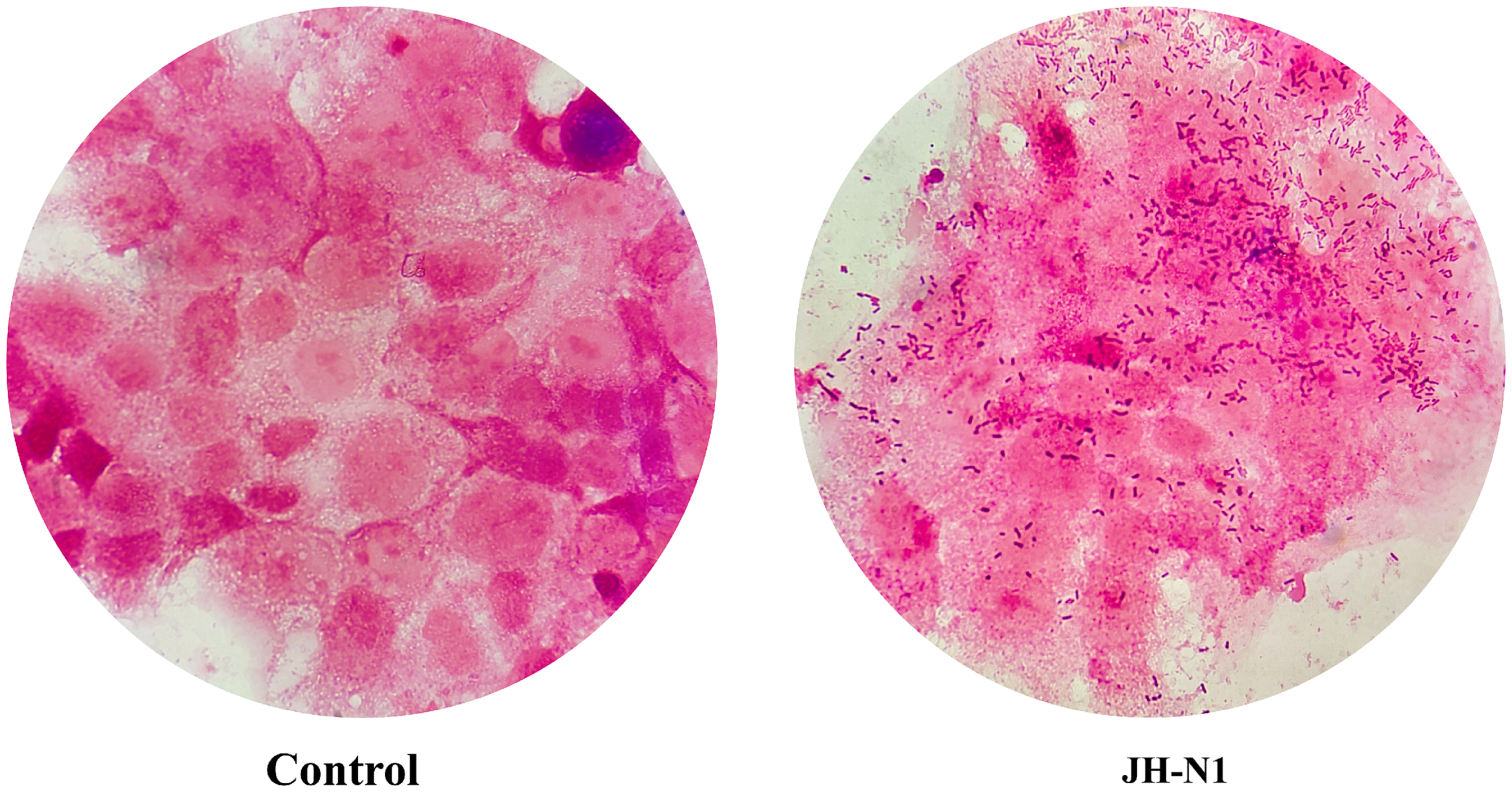
Adhesion of *L. plantarum* JH-N1 to Caco-2 cells. Representative microscopic image showing attachment of strain JH-N1 to Caco-2 cells after incubation.

Before the adhesion assay, *L. plantarum* JH-N1 and its CFS were separately incubated with Caco-2 cells for 12 h. No morphological alterations were observed during this period, indicating an absence of detectable cytotoxicity. According to the classification proposed by previous researcher (Reddy and Austin, 2017), the adhesive capacity of *Lactiplantibacillus* spp. is categorized as non-adhesive (NA, 0–10%), weakly adhesive (W, 10–20%), moderately adhesive (M, 20–50%), and strongly adhesive (S, >50%). *L. plantarum* JH-N1 exhibited strong adhesion, with an adhesion rate of 60.64% ± 4.27%. Attachment of *L. plantarum* JH-N1 to Caco-2 cells was further confirmed by microscopic observation (Figure 11).

#### 3.2.8 Susceptibility test

The antibiotic susceptibility profile of *L. plantarum* JH-N1 is presented in Supplementary Table S6. The results showed that *L. plantarum* JH-N1 was susceptible to most of the antibiotics tested, exhibited intermediate susceptibility to azithromycin, streptomycin, and gentamicin, and was resistant to polymyxin B, ciprofloxacin, and ofloxacin.

### 3.3 *In vivo* evaluation of its potential probiotic properties

#### 3.3.1 Acute toxicity tests

After confirming the beneficial effects of probiotics, *in vivo* safety assessments are imperative to accelerate their practical application. During the acute toxicity tests, no pathological symptoms were observed. The hair appearance remained unaltered, and they maintained good health and activity levels. Significantly, no instances of animal mortality were recorded, even in the high-dose group that received 1 × 10^11^ CFU of *L. plantarum* JH-N1 in mice. Furthermore, the presence of *L. plantarum* JH-N1 was not detected in the bloodstream.

#### 3.3.2 Subchronic toxicity tests

**Figure 12:**
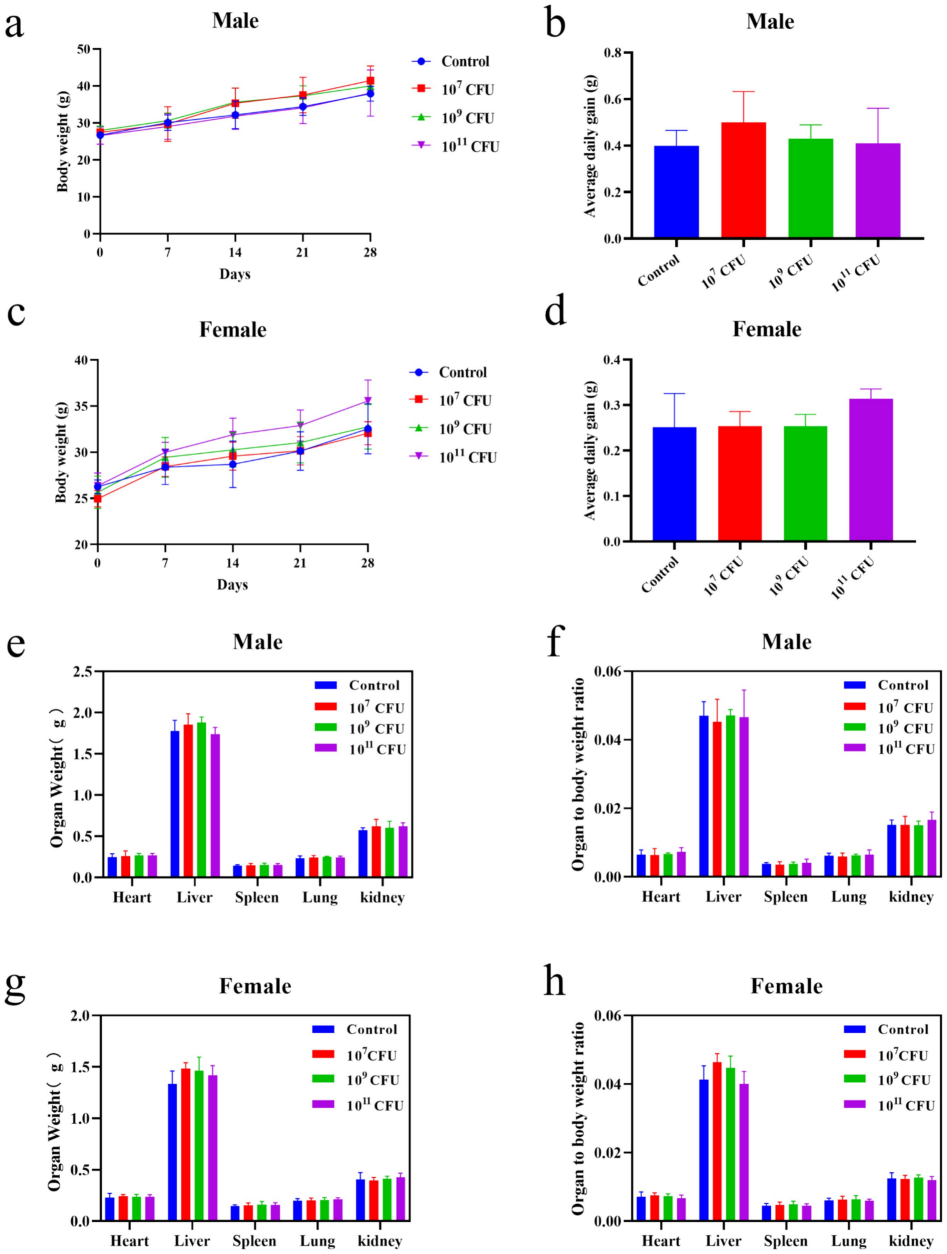
Effects of *L. plantarum* JH-N1 on body weight, daily weight gain, organ index, and organ-to-body weight ratio. **(a)** Body weight gain of male mice. **(b)** Daily weight gain of male mice. **(c)** Body weight gain of female mice. **(d)** Daily weight gain of female mice. **(e)** Organ index of male mice. **(f)** Organ-to-body weight ratio of male mice. **(g)** Organ index of female mice. **(h)** Organ-to-body weight ratio of female mice.

After confirming the beneficial effects of probiotics, *in vivo* safety assessments are imperative to facilitate their practical application. During the acute toxicity test, no pathological symptoms were observed. The coat appearance remained normal, and the animals maintained good health and activity throughout the study. Importantly, no mortality was recorded, even in the high-dose group receiving 1 × 10^11^ CFU of *L. plantarum* JH-N1. Furthermore, *L. plantarum* JH-N1 was not detected in the bloodstream of mice. Various clinical signs, including fur condition, eye appearance, behavioral patterns, tremors, twitches, lethargy, sleep, gait, and postural changes, were carefully monitored daily. Remarkably, no abnormalities were detected, and no mortality was recorded. In addition, the mice were weighed weekly. As shown in Figure 12, the body weight and daily weight gain of both male and female mice administered *L. plantarum* JH-N1 did not exhibit any significant changes. Importantly, no significant differences were observed in the organ indices or organ-to-body weight ratios of the heart, liver, spleen, lungs, and kidneys between the control and treated groups.

**Figure 13:**
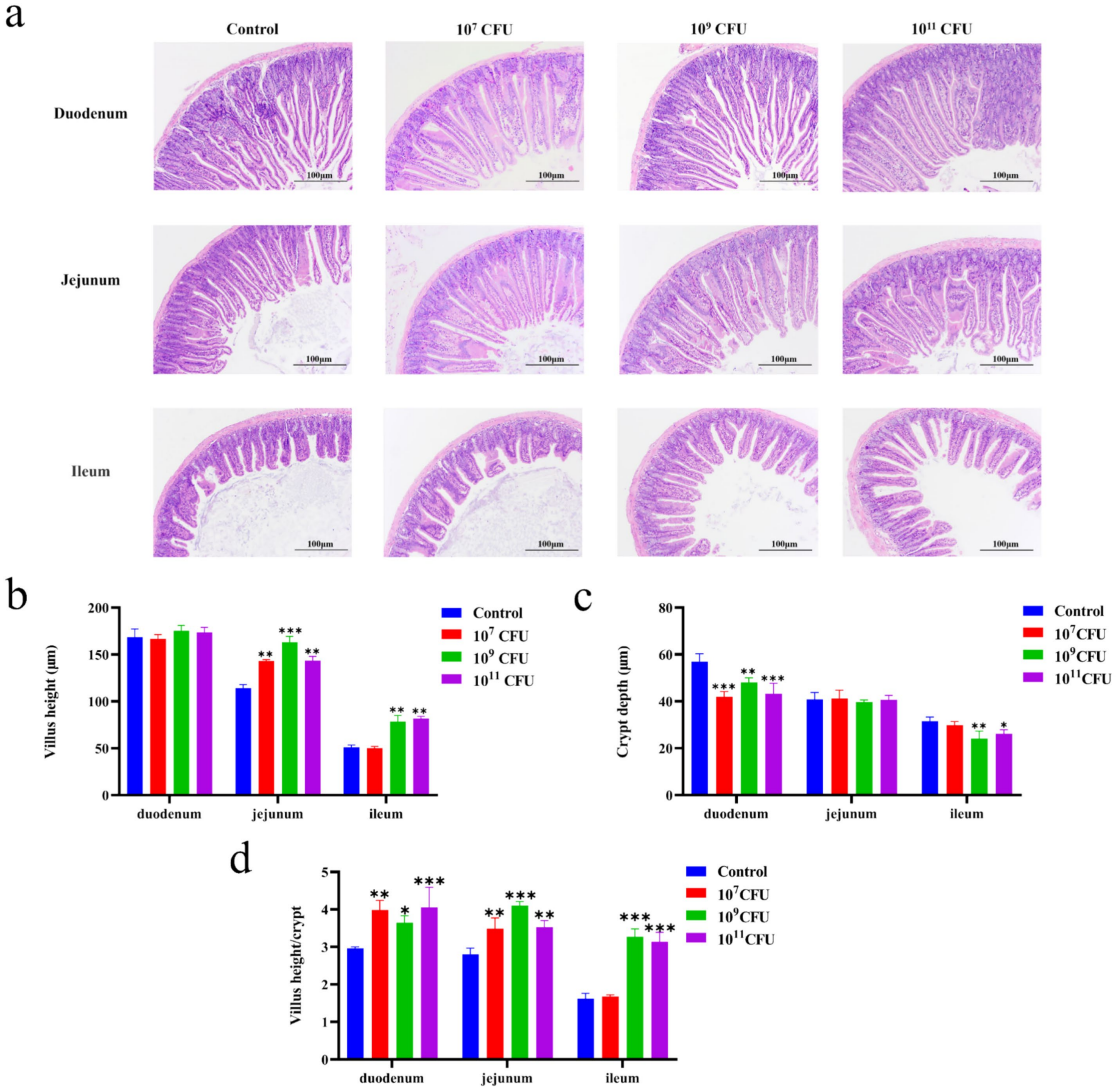
Effects of *L. plantarum* JH-N1 on intestinal histomorphology in mice. **(a)** Representative H&E-stained intestinal sections (×100). **(b)** Villus height. **(c)** Crypt depth. **(d)** Villus height-to-crypt depth ratio. *p < 0.05, **p < 0.01, ***p < 0.001.

Following euthanasia, the mice were subjected to cervical dislocation, and the heart, liver, spleen, lungs, and kidneys were collected and weighed. It is widely recognized that, even in the absence of gross morphological alterations between treated and control animals, differences in organ weight may occur; therefore, the organ index is an important parameter for evaluating strain safety. The organ weights obtained in the 28-day subchronic toxicity test are presented in Figure 12. Importantly, no significant differences were observed in the organ indices or organ-to-body weight ratios of the heart, liver, spleen, lungs, and kidneys between the control and treated groups. Cultures of the heart, liver, spleen, lungs, kidneys, and blood were negative for *L. plantarum* JH-N1. Furthermore, no significant alterations were observed in these organs, and no histopathological abnormalities were detected during the experimental period. These findings suggest that *L. plantarum* JH-N1 is safe for animal consumption at doses of up to 1 × 10^11^ CFU/animal/day. Examination of the intestinal tract revealed that the duodenum, jejunum, and ileum of both the control and treated groups exhibited well-preserved intestinal mucosal architecture, with villi arranged in an orderly and compact manner. *L. plantarum* JH-N1 significantly promoted villus growth in the jejunum and ileum, increased crypt depth in the duodenum and ileum, and resulted in significantly higher villus height-to-crypt depth ratios compared with the control group (Figure 13).

**Figure 14:**
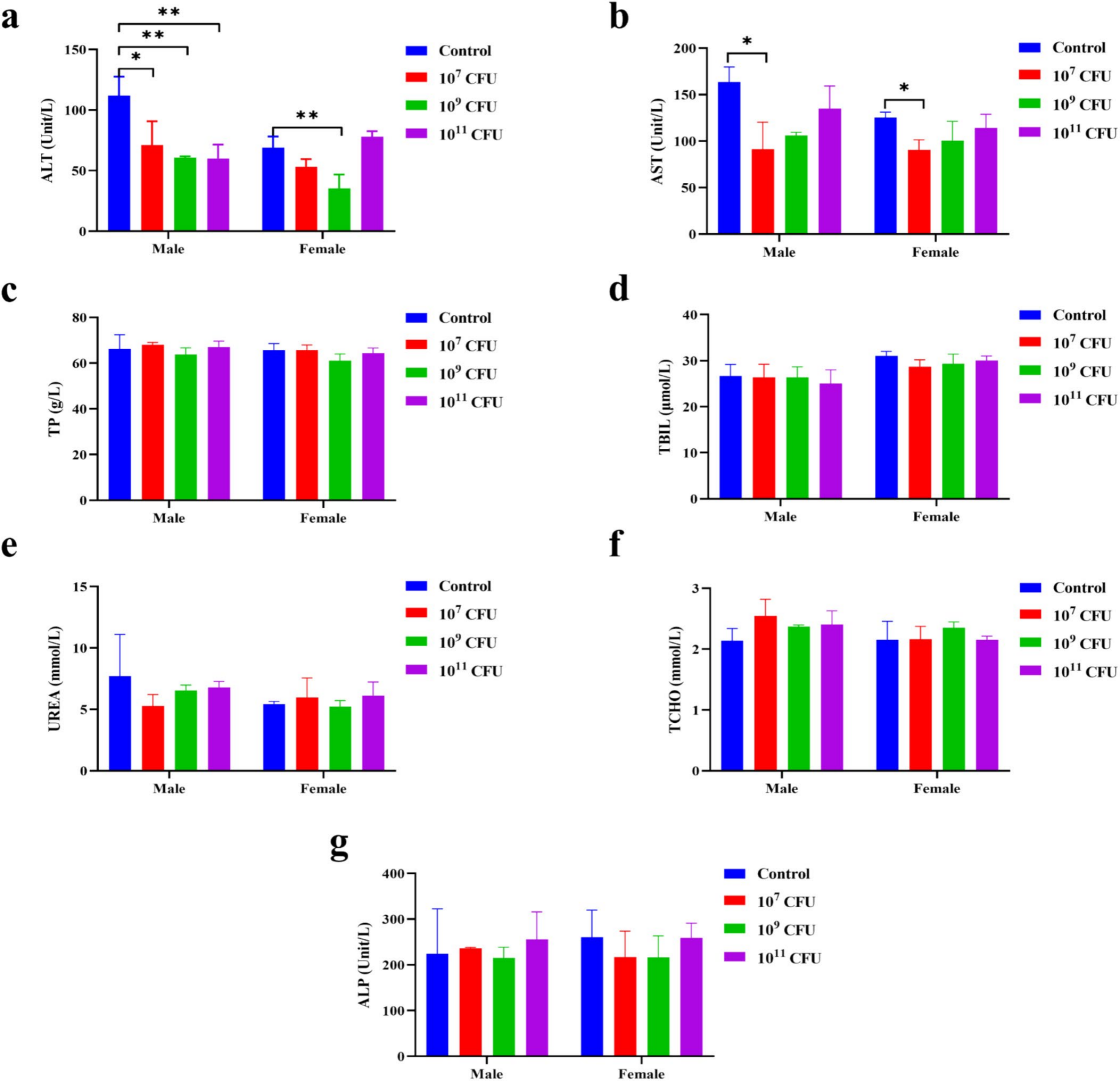
Effects of *L. plantarum* JH-N1 on blood biochemical parameters in mice. **(a)** Alanine aminotransferase (ALT). **(b)** Aspartate aminotransferase (AST). **(c)** Total protein (TP). **(d)** Total bilirubin (TBIL). **(e)** Urea. **(f)** Total cholesterol (TCHO). **(g)** Alkaline phosphatase (ALP). *p < 0.05, **p < 0.01.

Supplementary Table S7 presents the hematological findings, including hemoglobin, white blood cell count, red blood cell count, mean corpuscular hemoglobin concentration, mean corpuscular volume, and mean corpuscular hemoglobin, with no significant differences observed between the control and probiotic-treated groups. Conversely, *L. plantarum* JH-N1 significantly reduced (*p* < 0.05) serum transaminase levels, including alanine aminotransferase and aspartate aminotransferase, compared with the control group (Figures 14a,b). However, no significant alterations were detected in total protein, total bilirubin, urea, total cholesterol, or alkaline phosphatase in animals administered probiotics compared with the control group (Figures 14c–g).

#### 3.3.3 Intestinal flora sequencing results

**Figure 15:**
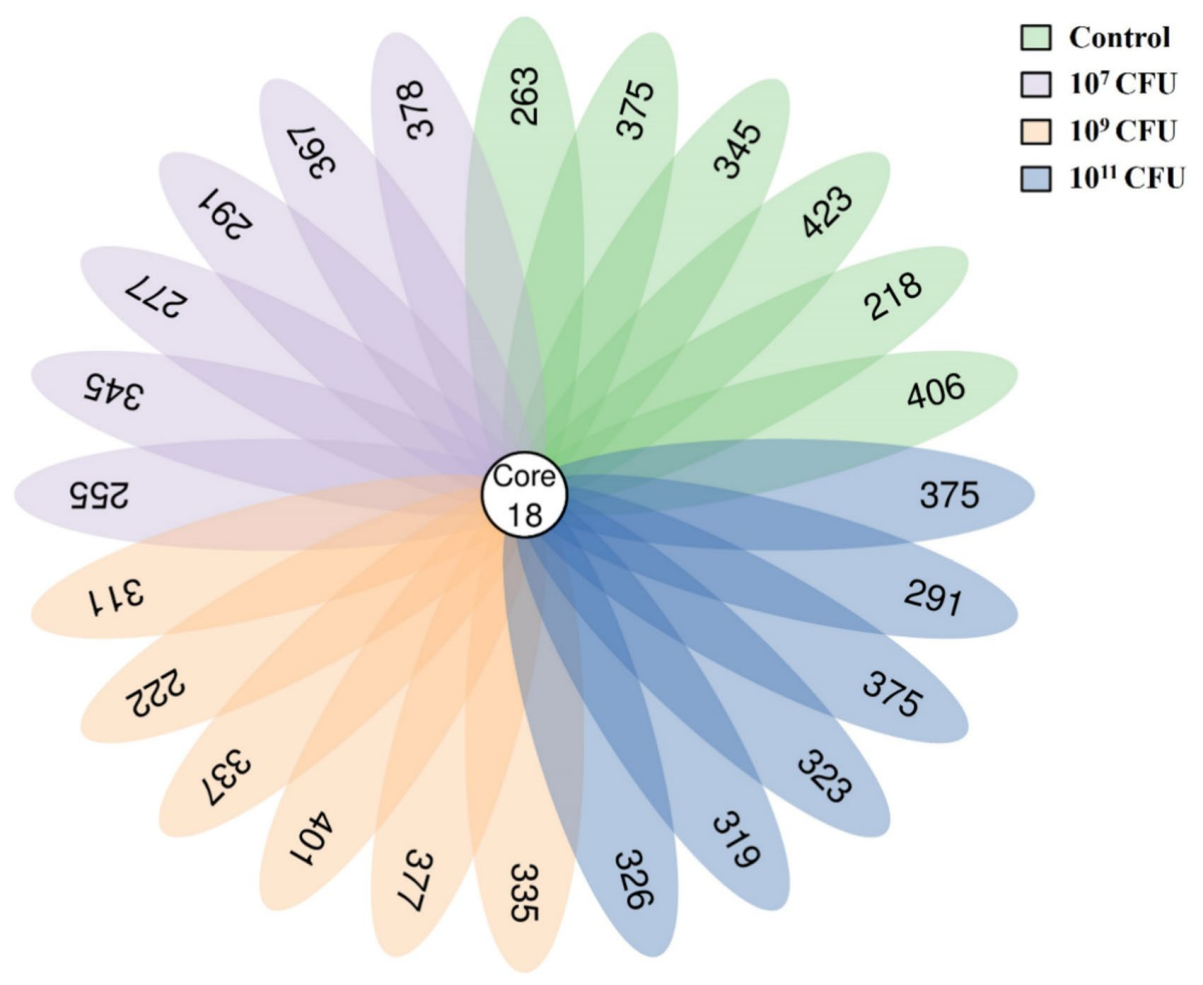
Differences in the distribution of ASVs showed by the Flower plot. Petals represent unique ASVs in each group, and the center indicates 18 ASVs shared among the control, 10^7^ CFU, 10^9^ CFU, and 10^11^ CFU groups.

The flower plot is mainly used to display the distribution of unique and common elements. The results were visualized in a flower plot, which facilitated comprehension of the ASV count within each sample and the number of ASVs shared among all samples. The control, 10^7^ CFU, 10^9^ CFU, and 10^11^ CFU groups exhibited 2,030, 1,913, 1,983, and 2,009 unique ASVs, respectively, with a total of 18 common ASVs among the four groups (Figure 15). The 18 shared ASVs are *Muribaculaceae*, *Helicobacter*, uncultured_bacterium, *Alloprevotella*, *Prevotellaceae*_UCG-001, *Muribaculaceae*, *Muribaculaceae*, *Muribaculaceae*, *Bacteroides*, *Rikenella*, *Rikenellaceae*_RC9_gut_group, *Bacteroides*, *Parabacteroides*, *Muribaculaceae*, *Lactobacillus*, *Muribaculaceae*, *Lactobacillus*, and *Muribaculum*.

Alpha diversity analysis provides a quantitative assessment of species richness and diversity within microbial communities. The Simpson, Chao1, and Ace indices are commonly used to estimate community richness and diversity, with higher values generally indicating greater richness and ecological complexity. No significant differences in alpha diversity were observed between the experimental and control groups (Supplementary Figure S4). Furthermore, comparison among the four groups indicated no marked changes in overall bacterial diversity, although a trend toward increased richness and evenness was observed, supporting the reliability of the sequencing results.

Principal coordinate analysis (PCoA) was used to evaluate differences in microbial community structure among groups. Each point represents an individual sample, and samples from the same group are shown in the same color. Greater proximity among samples within a group, together with separation from other groups, indicates stronger clustering and intergroup dissimilarity. PCoA revealed substantial overlap among the four groups in multivariate space (Supplementary Figure S5), suggesting that there is no significant difference in species richness between the groups.

**Figure 16:**
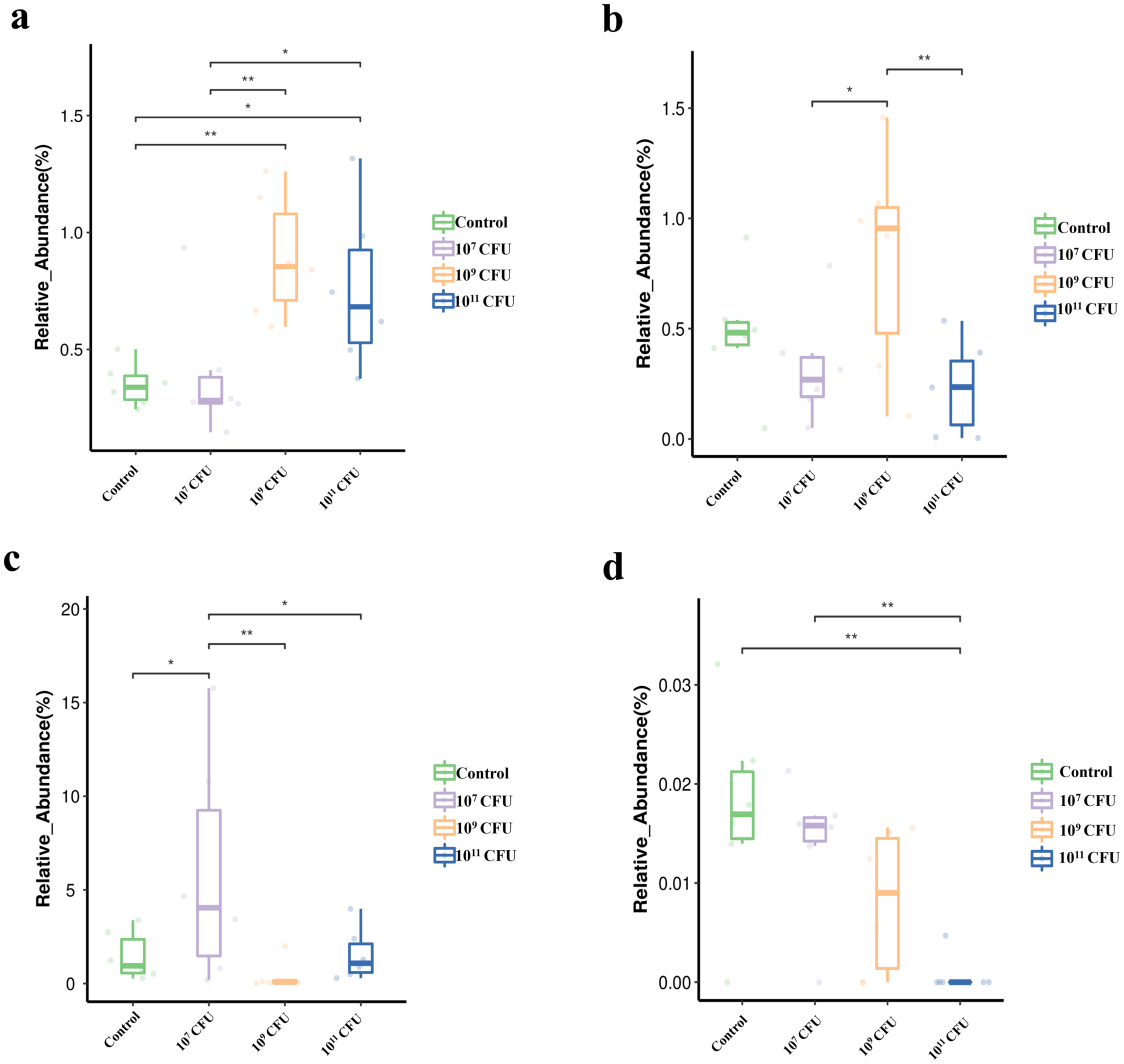
Differential species Boxplot. Relative abundance of representative differential taxa among groups. Enrichment of *Lactobacillus* **(a)**, *Clostridia vadin* BB60 **(b)**, and *Alloprevotella* **(c)** following *L. plantarum* JH-N1 administration. Reduced abundance of *Neisseria* **(d)** in the high-dose groups. *p < 0.05, **p < 0.01.

Statistical analyses were performed at multiple taxonomic levels, including phylum, family, genus, and species. Relative abundance boxplot analyses of the top differential taxa were conducted to identify dominant microbial changes within and between groups. The results showed that the relative abundance of *Lactobacillus* was significantly higher in mice receiving *L. plantarum* JH-N1 at 10^9^ and 10^11^ CFU than in the control group. In addition, the relative abundance of *Clostridia vadin* BB60 was significantly increased in the 10^9^ CFU group, whereas *Alloprevotella* was significantly enriched in the 10^7^ CFU group compared with the control group (Figures 16a–c). Moreover, at higher doses of *L. plantarum* JH-N1, the relative abundance of *Neisseria* was reduced and was significantly lower than that in the control group (Figure 16d).

## 4 Discussion

The increasing prevalence of bacterial antibiotic resistance presents a significant challenge in the fight against infectious diseases (Chlebicz and Śliżewska, 2018; Nwobodo et al., 2022). In recent years, probiotics have emerged as a promising alternative to antibiotics for controlling infections in animals and enhancing animal production. Several studies have investigated LAB with remarkable functional attributes as potential probiotics (Vinayamohan et al., 2024). *L. plantarum* JH-N1 was isolated from sapropel in an agronomy experimental field and demonstrated the highest antibacterial activity. Previous research has indicated that LAB metabolites, specifically hydrogen peroxide and acid, can inhibit the growth of various microorganisms (Reid, 2012). Additionally, LAB secretes various antimicrobial substances, including organic acids, hydrogen peroxide, and bacteriocins (Özogul and Hamed, 2018). Our results indicated that after excluding acid and hydrogen peroxide, *L. plantarum* JH-N1 still exhibited antibacterial activity. This suggests a high possibility of bacteriocins in the extracellular secretion of *L. plantarum* JH-N1. This finding is similar to the study of bacteriocin-producing *L. plantarum* ZJ5 isolated by Song et al. (2014) and Wang et al. (2018). Moreover, the cell-free supernatant of *L. plantarum* JH-N1 exhibited higher antibacterial activity against pathogens than that reported in earlier studies (Jabbari et al., 2017; Cao et al., 2019; Bhushan et al., 2021). The CFS of *L. plantarum* JH-N1 exhibits stronger antibacterial activity against *E. coli* and *S. typhimurium* than the *L. plantarum* BBC32A, which was isolated by Bhushan et al. (2021). Its antibacterial activity was twice as strong as that of *L. plantarum* STDA10 isolated by Cao et al. The antibacterial activity of CFS from *L. plantarum* JH-N1 against *S. typhimurium* and *S. aureus* is twice that of *L. plantarum* FB003 isolated by Jabbari et al. Most importantly, antiSMASH was utilized to predict the secondary metabolites of *L. plantarum* JH-N1. A segment of its gene showed high similarity to the bacterial plantaricin E. Anderssen et al. have isolated and purified bacteriocin plantaricin E, demonstrating its antibacterial activity (Anderssen et al., 1998). This indicates that *L. plantarum* JH-N1 indeed contains antibacterial substances similar to bacteriocins. However, the present study failed to fully clarify the antibacterial properties of *L. plantarum* JH-1 as well as the molecular action mechanism of plantaricin EF. Further mechanistic investigations of the bacteriocins derived from this strain will be implemented in subsequent research.

Whole-genome analysis showed that JH-N1 possesses a typical *L. plantarum* genome architecture, with a chromosome size (~3.2 Mb) and GC content (44.5%) consistent with previously reported strains (Nikodinoska et al., 2022). The large and flexible genome of *L. plantarum* is thought to facilitate adaptation to diverse ecological niches, including fermented foods and animal gastrointestinal tracts (Iarusso et al., 2025). COG categorized all predicted proteins, with unknown/general function prediction dominating, followed by carbohydrate transport and metabolism (Supplementary Figure S6a). CAZy annotation targeting carbohydrate-active enzymes uncovered 73 relevant genes, dominated by glycosyltransferases then glycoside hydrolases (Supplementary Figure S6b). KEGG annotation results reveal that most enriched genes fall into metabolic pathways dominated by carbohydrate metabolism (Supplementary Figure S6c), followed by genetic information processing pathways. GO annotation results display that enriched genes primarily relate to metabolic and cellular processes in biological process, while catalytic activity ranks top among molecular function terms (Supplementary Figure S6d), followed by binding and transporter activity. Notably, JH-N1 harbored two putative class IIb bacteriocin genes with high sequence identity to plantaricin EF, suggesting a genetic basis for its strong antibacterial activity, as plantaricins are among the best-characterized antimicrobial peptides produced by *L. plantarum* (Heeney et al., 2019). Genome mining also detected no confirmed virulence determinants, prophages or genomic islands associated with pathogenicity. In addition, multiple genes related to acid, bile, oxidative and temperature stress tolerance were identified, consistent with the strong *in vitro* stress resistance of JH-N1. Together with its high hydrophobicity, auto-aggregation, co-aggregation and epithelial adhesion capacity, these traits suggest that JH-N1 is well adapted for gastrointestinal survival, transient colonization and competitive exclusion of pathogens.

Despite the generally recognized safety of LAB, evaluating the safety of this specific strain for its intended production and use is crucial. The primary criteria for assessing the strain’s safety involve examining its hemolytic and mucin-degrading activity (Palaniyandi et al., 2017). In the hemolysis experiment, *L. plantarum* JH-N1 did not exhibit hemolytic properties. LAB typically shows no hemolytic activity, which enhances the safety of the strain (Adimpong et al., 2012). During the mucin degradation assay, the absence of mucin degradation activity indicates that the strain does not harm the protective mucus layer in the gastrointestinal tract. This ensures the integrity of the body’s mucosal defense system, further reflecting the safety of the strain (Zhou et al., 2001). These results are consistent with the data reported in earlier studies (Abe et al., 2010; Aristimuño Ficoseco et al., 2018). To further evaluate safety, the viability of Caco-2 cells in the presence of *L. plantarum* JH-N1 and CFS was examined. *L. plantarum* JH-N1 infected cells at different MOIs with no significant change in cell viability. Similar results were observed for the non-toxic cell supernatant, which exhibited a promoting effect on cellular proliferation at concentrations of 5 and 10%. This confirms the safety of *L. plantarum* JH-N1 and supports subsequent adhesion tests of this strain to Caco-2 cells.

*Lactiplantibacillus* spp. can appreciably tolerate the adversities of the gastrointestinal tract, which is a prerequisite condition for the efficacy and viability of probiotics. The acid and bile salt tolerance to *L. plantarum* JH-N1 is stronger than that of other *Lactiplantibacillus* spp. reported in previous studies (Tang et al., 2018; Rastogi et al., 2020; Zheng et al., 2020). The survival rate of *L. plantarum* JH-N1 incubated at pH 2 for 4 h is 54%, while the survival rate of *L. plantarum* MA2 at pH 2.5, as reported by Tang et al. (2018), was completely lost. The survival rate of *L. plantarum* JH-N1 at a bile salt concentration of 0.3% is over 92%, which is stronger than *L. mucosae* SRV10, as reported by Rastogi et al. (2020). After 4 h at a 0.3% bile salt concentration, the survival rate of *L. plantarum* JH-N1 was 86%, while Tang et al. (2018) reported that *L. plantarum* MA2 had a survival rate of only 50%. Moreover, Zheng et al. (2020) reported that the survival rate of *L. plantarum* E680 after 3 h at a 0.3% bile salt concentration was 80%, indicating that its bile salt tolerance is lower than that of our strain. Additionally, *L. plantarum* JH-N1 has a survival rate of up to 63% at 1% bile salt concentration for 4 h. Subsequently, sequencing analysis was conducted on the complete genome of *L. plantarum* JH-N1, which revealed the presence of the bile salt resistance gene *cbh* and pH resistance-related genes, including *clcA*, ClcA, atpB, atpE, atpF, atpH, atpA, atpG, atpD, atpC, and nhaC_2. This finding is consistent with earlier reports on the analysis of probiotic resistance genes (Wang et al., 2023). This does not fully explain why *L. plantarum* JH-N1 shows greater tolerance to acid and bile salts than these LAB strains. Future work will focus on elucidating this mechanism.

Current evidence suggests that the cell surface hydrophobicity is related to bacterial adhesion (Salas-Tovar et al., 2021). The study by Doyle et al. also has indicated that the hydrophobicity of bacterial surfaces promotes bacteria colonization in intestinal epithelial tissue (Doyle, 2000). The cell surface hydrophobicity of *L. plantarum* JH-N1 was superior to that of two intestinal pathogenic bacteria. These research results are consistent with findings from earlier studies (Rastogi et al., 2020). In addition to cell surface hydrophobicity, assessing the auto-aggregation and copolymerization capabilities of LAB strains with high adhesion capacity is essential. The ability to aggregate is a crucial characteristic of probiotics, contributing to the host’s defense against pathogen colonization (Barzegar et al., 2021). The auto-aggregation ability of *L. plantarum* JH-N1 and co-aggregation ability against four common pathogenic bacteria were both above 60%, which is consistent with previous findings (Xu et al., 2009). In the adhesion assay, *L. plantarum* JH-N1 exhibited strong adhesion ability to Caco-2 cells, recording a percentage of 60.64% ± 4.27%. This result is stronger than that reported in a previous study (Jabbari et al., 2017). Jabbari et al. (2017) determined the adhesion rate of *L. plantarum* FB003 to Caco-2 as 35%. In light of the experimental data on hydrophobicity, auto-aggregation, co-aggregation, and adhesion, this suggests that *L. plantarum* JH-N1 possesses exceptional adhesion capabilities. Its complete genome was analyzed and found to contain the *epsh* gene (Xiao et al., 2023), an adhesion-related gene, but the exact relationship remains to be investigated. *L. plantarum* JH-N1 exhibited susceptibility to most antibiotics, moderate susceptibility to aminoglycosides, and resistance to polypeptides and quinolones, aligning with findings from the antibiotic sensitivity test conducted on *L. plantarum* ZDY 2013 (Huang et al., 2015). Moreover, our preliminary findings indicate that *L. plantarum* JH-N1 can effectively inhibit infection by multiple viruses *in vitro*, including PEDV, PDCoV, and feline infectious peritonitis virus (data not shown). Xing et al. (2022) reported that *L. plantarum* 0111 confers significant protection against H9N2 influenza virus infection by enhancing antiviral immunity and modulating the gut microbiota, further supporting the broad antiviral potential of *L. plantarum*. Similarly, Lu et al. (2024) showed that *L. plantarum* GUANKE protects mice against influenza infection by reducing viral load and enhancing dendritic cell-mediated antiviral immunity, further underscoring the antiviral capacity of *L. plantarum*. Further investigations into the underlying antiviral mechanisms and translational potential of *L. plantarum* JH-N1 are currently underway.

The strain demonstrated exceptional antimicrobial activity and probiotic properties during *in vitro* tests, along with preliminary safety assessments. However, to fully ascertain its immunogenicity, toxicity, and practical significance, *in vivo* safety evaluations are essential (Sahoo et al., 2017). Consequently, acute and subacute toxicity tests on mice were conducted to assess the safety of orally administered *L. plantarum* JH-N1. Consistent with previously published data, the acute toxicity test showed that mice exposed to various doses of the strain did not exhibit any disease symptoms or detectable levels of *L. plantarum* JH-N1 in blood (Zhou et al., 2000). During the subchronic toxicity study, no abnormal clinical symptoms or deaths were observed. There was no significant difference in weight and organ index among the different groups. *L. plantarum* JH-N1 is not detected in mouse blood and organs, further confirming the safety of this strain. Surprisingly, *L. plantarum* JH-N1 significantly promoted the growth of jejunal and ileal villi, deepened the depth of crypts in the duodenum and ileum, and resulted in significantly higher ratios of intestinal villus lengths to crypt depths compared to those of the control group. Hematological parameters are commonly used to assess inflammatory responses and hematologic disorders, while blood biochemistry evaluations help identify organ-related issues (Petterino and Argentino-Storino, 2006). During the subacute toxicity experiment, no significant changes in hematological parameters were observed between the experimental group and the control group. These findings are consistent with previous studies on *L. plantarum* MTCC 5690 and *L. fermentum* MTCC 5689 (Pradhan et al., 2019). Blood biochemistry experiments on mice revealed a significant decrease in serum transaminases, which can be attributed to the specific dosage effect of probiotics. This finding aligns with the results of Samtiya et al. (2020). These results further confirm that *L. plantarum* JH-N1 is not only safe but also has probiotic effects, which are consistent with previous safety assessments of probiotics (Kim et al., 2017; Wang et al., 2019; Zulkhairi Amin et al., 2023).

The intestinal flora, consisting of beneficial, harmful, and neutral bacteria, constitutes the most extensive micro-ecosystem within the human body (Ma et al., 2019). After administering *L. plantarum* JH-N1 to mice and analyzing their fecal samples through Alpha and Beta Diversity analysis, it was observed that there was no significant difference between the control and experimental groups, suggesting that *L. plantarum* JH-N1 has a limited impact on the diversity of the mice’s intestinal flora. The results of the species analysis of inter-group differences in mouse gut microbiota indicated that *L. plantarum* JH-N1 caused a significant increase in the relative abundance of beneficial bacteria (*Lactobacillus*, *Allopreivotella*, and *Clostridia vadin* BB60) and a significant decrease in the relative abundance of deleterious bacteria (*Neisseria*) in the mice gut microbiota (Song et al., 2023; Zhu et al., 2021). The 18 shared ASVs indicates that oral administration of *L. plantarum* JH-N1 does not disrupt the foundational intestinal microbial homeostasis in mice. The majority of these taxa fall within the carbohydrate-degrading phylum *Bacteroidota*, with multiple ASVs assigned to *Muribaculaceae* and *Muribaculum* dominating the core microbiota. These microbes are specialized in fermenting dietary fiber and intestinal mucin to generate propionate and acetate, which in turn sustain intestinal barrier integrity and confer resistance against pathogen colonization. *Bacteroides*, *Parabacteroides*, *Rikenellaceae* RC9 gut group, *Alloprevotella*, and *Prevotellaceae* UCG-001 act synergistically to stabilize carbohydrate metabolism and sustain basal immune homeostasis. Two shared *Lactobacillus* ASVs represent native murine autochthonous lactobacilli, forming a baseline beneficial pool alongside exogenous JH-N1. The single shared *Helicobacter* ASV represents a low-abundance commensal strain that exhibits no pathogenic traits in healthy mice. Collectively, this fixed core flora maintains basal gut metabolic and immune function regardless of *L. plantarum* JH-N1 dosage variation.

In conclusion, the *L. plantarum* JH-N1 isolated from crop soil in the field of agriculture possesses various antibacterial properties and demonstrates good safety as well as significant probiotic potential *in vitro* and *in vivo*. Based on the results obtained, the *in vivo* mechanism of action of *L. plantarum* JH-N1 was predicted; it can colonize the intestinal tract due to its excellent environmental tolerance, adhesion, and cell surface hydrophobicity. It also utilizes copolymerization to bind pathogenic bacteria or secrete antibacterial substance to kill pathogenic bacteria, thereby preventing their colonization in the intestine. The absence of hemolytic activity and mucin-degrading activity ensures the integrity of the intestinal barrier and promotes the growth of intestinal villi (Supplementary Figure S7).

## 5 Conclusion

In summary, *L. plantarum* JH-N1 is a novel sapropel-derived strain that exhibits antimicrobial activity, favorable genomic safety characteristics, strong gastrointestinal stress tolerance, and excellent adhesion-related probiotic traits. *In vitro* and *in vivo* evaluations confirmed its safety, with no evidence of cytotoxicity, haemolysis, or adverse effects in mice. Importantly, oral administration of JH-N1 improved intestinal morphology and beneficially modulated the gut microbiota. Collectively, these findings identify JH-N1 as a promising probiotic candidate for veterinary and biomedical applications, although further studies are needed to elucidate its molecular mechanisms and translational potential.

## Data Availability

The datasets presented in this study can be found in online repositories. The names of the repository/repositories and accession number(s) can be found in the article/Supplementary material.

## Footnotes

1
http://www.cbs.dtu.dk/services/TMHMM/

2
http://www.cbs.dtu.dk/services/SignalP

3
https://swissmodel.expasy.org/
