# Supplementary material for: A sapropel-derived Lactiplantibacillus plantarum with antimicrobial activity improves intestinal architecture and remodels gut microbiota
Source: Front Microbiol. 2026 Jul 30;17:1881881. doi: 10.3389/fmicb.2026.1881881 (PMC13469455; doi:10.3389/fmicb.2026.1881881)
Supplement: Supplementary file 1 [file Data_Sheet_1.DOCX]

**Table S1** Bacterial strains and their culture condition in this study.

| **Strain** | **Medium** | **Temperature (℃)** | **Condition** |
| --- | --- | --- | --- |
| *Staphylococcus aureus* ATCC 25923 | TSB | 37 | aerobic |
| *Listeria monocytogenes* CICC 21634 | TSB | 37 | aerobic |
| *Escherichia coli* ATCC 25922 | LB | 37 | aerobic |
| *Salmonella Typhimurium* SL1344 | LB | 37 | aerobic |
| Candidate strain | MRS | 37 | anaerobic |

TSB and MRS (Qingdao Hope Bio-Technologyc Co., Ltd, Shandong, China); 1 g of NaCl (Hangzhou Biological Reagent Co., Ltd. Zhejiang, China ), 1 g of trypsin (Aoboxing Biotechnology Co., Ltd., Beijing, China), and 0.5 g of yeast extract powder (Thermo Fisher Scientific, Shanghai, China) were required to prepare 100 mL of LB.

**Table S2** Antibacterial activity of the candidate strains from soil samples against pathogenic bacteria was assessed using the Oxford cup agar diffusion method.

| **Source** | **Strain** | **Zone of inhibition (mm)** | | | |
| --- | --- | --- | --- | --- | --- |
|  |  | ***Staphylococcus aureus*** | ***Listeria monocytogenes*** | ***Escherichia***  ***coli*** | ***Salmonella Typhimurium*** |
| Rapeseed flower root soil | N01 | + | ++ | ++ | + |
|  | N02 | - | - | + | - |
| Forest Rhizosphere soil | N03 | - | + | ++ | ++ |
|  | N04 | - | + | + | + |
| Creek silt | N05 | - | ++ | ++ | + |
|  | N06 | + | ++ | + | + |
| Lake bottom sludge | N07 | ++ | + | - | + |
|  | N08 | + | + | + | ++ |
|  | N09 | + | ++ | - | - |
| Agronomy experimental field crop soil | JH-N1 | +++ | ++ | +++ | ++ |
|  | N11 | ++ | + | + | + |
|  | N12 | - | ++ | ++ | ++ |
|  | N13 | - | - | +++ | ++ |
|  | N14 | + | ++ | + | ++ |
|  | N15 | ++ | ++ | + | + |

The antibacterial activity of the candidate strains isolated from samples was expressed as (-) no inhibition (＜12mm), (+) weak inhibition (12–14 mm), (++)intermediate inhibition (14–16 mm), (+++) strong inhibition (＞16mm).

**Table S3** Toxicity factor annotation.

| **VFDB A** | **Gene ID** | **VFG**  **Symbol** | **VF Info** | **VF Name** | **VF**  **term** |
| --- | --- | --- | --- | --- | --- |
| VFG000077 | ctg00003_01606 | *clpP* | ATP-dependent Clp protease  proteolytic subunit | clpP | VF0074 |

**Table S4** Drug resistance function annotation.

| **CARD** | **GeneID** | **ARO Name** | **Gene Family** | **Resistance Mechanism** | **CARD Short Name** |
| --- | --- | --- | --- | --- | --- |
| ARO: 3000024 | ctg00005_ 02115 | *patA* | ATP-binding cassette (ABC) antibiotic efflux pump | antibiotic efflux | patA |
| ARO: 3000025 | ctg00002_ 00868 | *patB* | ATP-binding cassette (ABC) antibiotic efflux pump | antibiotic efflux | patB |
| ARO: 3000074 | ctg00001_ 00004; ctg00005_ 02100 | *emrB* | major facilitator superfamily (MFS) antibiotic efflux pump | antibiotic efflux | emrB |
| ARO: 3000118 | ctg00005_ 02128 | *vgaB* | vga-type ABC-F protein | antibiotic target protection | vgaB |

**Table S5** Tolerance related genes of *L. plantarum* JH-N1.

| **Gene ID** | **Gene name** | **Gene function** |
| --- | --- | --- |
| **Universal stress family protein** | | |
| ctg00001_00018 | - | Putative universal stress protein |
| ctg00001_00319 | - | Putative universal stress protein |
| ctg00001_00357 | - | Putative universal stress protein |
| ctg00004_01994 | - | Putative universal stress protein |
| ctg00005_02106 | - | Putative universal stress protein |
| ctg00005_02193 | - | Putative universal stress protein |
| ctg00006_02261 |  | Putative universal stress protein |
| ctg00006_02340 | - | Putative universal stress protein |
| ctg00007_02478 | - | Putative universal stress protein |
| ctg00011_02867 | *yugI* | General stress protein 13 |
| **Heat stress resistance** | | |
| ctg00001_00588 | *dnaJ* | Chaperone DnaJ |
| ctg00001_00589 | *dnaK* | Chaperone DnaK |
| ctg00001_00590 | *GrpE* | Protein GrpE |
| ctg00001_00591 | *hrcA* | Heat-inducible transcription repressor HrcA |
| ctg00002_00846 | *hslO* | Hsp33 family molecular chaperone |
| ctg00007_02361 | *ctsR* | family transcriptional regulator |
| **Cold-shock stress resistance** | | |
| ctg00002_01269 | *cspL* | Cold shock protein 2 |
| ctg00007_02474 | *cspA* | Cold shock protein 1 |
| ctg00012_02909 | *cspLA* | Cold shock-like protein CspLA |
| **Bile salt resistance** | | |
| ctg00002_01367 | *cbh* | choloylglycine hydrolase |
| **pH stress resistance** | | |
| ctg00002_01185 | - | Acid shock protein |
| ctg00003_01734 | - | Alkaline shock protein 23 |
| ctg00003_01735 | - | Alkaline shock protein 23 |
| ctg00003_01615 | *ClcA* | H(+)/Cl(-) exchange transporter ClcA |
| ctg00004_01968 | *atpB* | ATP synthase subunit a |
| ctg00004_01969 | *atpE* | ATP synthase subunit c |
| ctg00004_01970 | *atpF* | ATP synthase subunit b |
| ctg00004_01971 | *atpH* | ATP synthase subunit delta |
| ctg00004_01972 | *atpA* | ATP synthase subunit alpha |
| ctg00004_01973 | *atpG* | ATP synthase gamma chain |
| ctg00004_01974 | *atpD* | ATP synthase subunit beta |
| ctg00004_01975 | *atpC* | ATP synthase epsilon chain |
| ctg00010_02783 | *nhaC_2* | Na+/H+ antiporter NhaC |
| **Oxidative stress** | | |
| ctg00001_00117 | *npr_1* | NADH peroxidase |
| ctg00001_00294 | *fabG_1* | 3-oxoacyl-[acyl-carrier-protein] reductase |
| ctg00001_00295 | *fabF* | 3-oxoacyl-[acyl-carrier-protein] synthase 2 |
| ctg00001_00301 | *fabI* | Enoyl-[acyl-carrier-protein] reductase [NADH] |
| ctg00002_00919 | *mvaA* | 3-hydroxy-3-methylglutaryl-coenzyme A reductase |
| ctg00002_00967 | *glpO* | Alpha-glycerophosphate oxidase |
| ctg00002_00981 | *mco* | Multicopper oxidase mco |
| ctg00002_01089 | *trxA_1* | Thioredoxin |
| ctg00002_01104 | *bsaA* | Glutathione peroxidase BsaA |
| ctg00002_01161 | *fabG_2* | 3-oxoacyl-[acyl-carrier-protein] reductase FabG |
| ctg00002_01339 | *katA* | Vegetative catalase |
| ctg00002_01442 | *trxA_2* | Thioredoxin |
| ctg00003_01586 | *trxB* | Thioredoxin reductase |
| ctg00004_01801 | *npr_2* | NADH peroxidase |
| ctg00006_02350 | *ytpP* | Thioredoxin-like protein YtpP |
| ctg00011_02832 | *trxA_3* | Thioredoxin |

**Table S6** Antibiotic resistance for *L. plantarum* JH-N1.

| **Kind** | **Antibiotic** | **Judgment criteria (mm)**  **R: Resistance; I: Intermediary; S: Sensitivity** | | | **Lactobacillus plantarum JH-N1** |
| --- | --- | --- | --- | --- | --- |
| Tetracyclines | Tetracycline | ≤11 | 12-14 | ≥15 | S |
| Chloram phenicols | Chloramphenicol | ≤12 | 13-17 | ≥18 | S |
|  | Florfenicol | ≤12 | 13-15 | ≥16 | S |
| Macrolides antibiotics | Erythromycin | ≤13 | 14-22 | ≥23 | S |
|  | Azithromycin | ≤13 | 14-17 | ≥18 | I |
| Aminoglycosides | Streptomycin | ≤14 | 15-18 | ≥19 | I |
|  | Gentamicin | ≤14 | 15-18 | ≥19 | I |
| β-Lactams | Ampicillin | ≤13 | 14-16 | ≥17 | S |
|  | Carbenicillin | ≤13 | 14-16 | ≥17 | S |
|  | Augmentin | ≤10 | 11-19 | ≥20 | S |
|  | Cefamandole | ≤14 | 15-17 | ≥18 | S |
|  | Ceftriaxone | ≤13 | 14-20 | ≥21 | S |
| Sulfonamides | Trimethoprim | ≤10 | 11-15 | ≥16 | S |
|  | Paediatric compound sulfamethoxazole tablets | ≤10 | 11-15 | ≥16 | S |
| Polypeptide | Polymyxin B | ≤8 | 9-11 | ≥12 | R |
| Furazolones | Furazolidone | ≤14 | 15-16 | ≥17 | S |
| Quinolones | Ciprofloxacin | ≤15 | 16-20 | ≥21 | R |
|  | Ofloxacin | ≤12 | 13-16 | ≥17 | R |

**Table S7** Effect of feeding *L. plantarum* JH-N1 on hematological parameters of mice.

| **Hematological Parameters** | **Control** | | **10^7^ CFU** | | **10^9^ CFU** | | **10^11^ CFU** | |
| --- | --- | --- | --- | --- | --- | --- | --- | --- |
|  | **Male** | **Female** | **Male** | **Female** | **Male** | **Female** | **Male** | **Female** |
| Hb (g/dL) | 13.96±2.70 | 15.88±0.45 | 14.88±1.59 | 14.88±1.45 | 15.02±1.71 | 15.60±2.30 | 14.08±3.02 | 15.84±1.02 |
| WBC (103) cells /mm3) | 8.22±2.17 | 7.63±2.84 | 6.58±1.01 | 7.96±2.07 | 7.92±2.79 | 8.71±1.73 | 7.36±1.09 | 7.57±0.54 |
| RBC (106) cells/mm3) | 5.94±3.31 | 4.43±2.12 | 6.90±2.68 | 4.45±0.49 | 6.19±2.14 | 4.73±2.02 | 6.06±2.40 | 6.30±1.36 |
| MCHC (g/dL) | 61.14±1.52 | 64.20±3.35 | 61.18±3.43 | 65.00±1.23 | 62.96±1.08 | 63.32±3.14 | 62.14±2.96 | 65.84±2.69 |
| MCV (FL) | 27.2±3.34 | 29.10±1.23 | 28.26±2.54 | 29.12±0.84 | 29.12±2.28 | 28.36±1.60 | 26.90±3.51 | 29.12±1.43 |
| MCH (pg) | 16.58±2.16 | 18.62±1.63 | 17.22±1.99 | 18.86±0.47 | 18.26±1.47 | 17.92±1.63 | 16.68±2.73 | 19.12±1.36 |

Values were presented as the mean ± standard deviation. Hb, hemoglobin; WBC, white blood cells count; RBC, red blood cell count; MCHC, mean corpuscular hemoglobin concentration; MCV, mean corpuscular volume; MCH, mean corpuscular hemoglobin.

**Figure S1.** Isolation and identification of antibacterial LAB.

**Figure S2.** Electropherogram of 16SrRNA gene after PCR of genomic DNA of JH-N1 strain. M: DNA Marker. 1: Amplification products of 16S rRNA gene of JH-N1 strain.

**Figure S3.** Phylogenetic and homology analyses of *L. plantarum* JH-N1. (**a**) Neighbor-joining phylogenetic tree of the JH-N1 strain. Numbers in parentheses indicate accession numbers of the reference strains. Values at branch nodes represent bootstrap confidence levels, and the scale bar indicates evolutionary distance. (**b**) Pie chart showing the homology distribution of *L. plantarum* JH-N1 based on genomic sequence comparison. (**c**) Maximum-likelihood phylogenetic tree constructed using full-length 16S rRNA gene sequences of *L. plantarum* JH-N1 and related strains.

**Figure S4.** Alpha-diversity.

**Figure S5.** PCoA analysis.

**FIGURE S6.** Functional annotation of the *L. plantarum* JH-N1. (**a**) COG functional classification analysis. (**b**) CAZy annotation of carbohydrate-active enzymes. (**c**) KEGG pathway enrichment and metabolic pathway analysis. (**d**) GO functional annotation analysis.

**Figure S7.** Predictive map of the role of *L. plantarum* JH-N1 in the intestine.
